# A Crystalline NiX_6_ Complex

**DOI:** 10.1021/jacs.4c12125

**Published:** 2024-12-13

**Authors:** Josef T. Boronski, Agamemnon E. Crumpton, Simon Aldridge

**Affiliations:** † Chemistry Research Laboratory, Department of Chemistry, Oxford OX1 3TA, U.K.; ‡ Department of Chemistry, Molecular Sciences Research Hub, Imperial College London, 82 Wood Lane, White City, London W12 7TA, U.K.

## Abstract

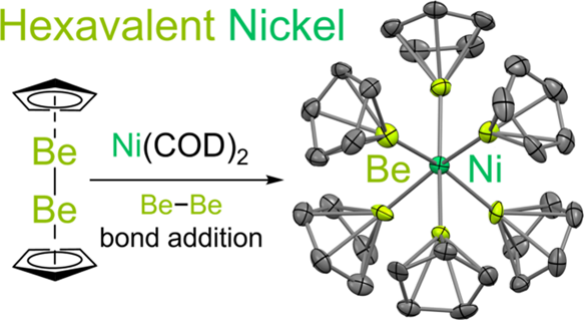

High-valent nickel species are implicated as intermediates in industrially relevant chemical transformations and in the catalytic cycles of metalloenzymes. Although a small number of tetravalent NiX_4_ complexes have been crystallographically characterized, higher nickel valence states have not been identified. Here we report a stable, crystalline NiX_6_ complex, Ni(BeCp)_6_ (**1**; cyclopentadienyl anion (Cp)), formed by the insertion of zerovalent nickel into three Be–Be bonds. This 16-electron species features an inverted ligand field, is diamagnetic, and exhibits *C*_3*v*_ symmetry, on account of the lifting of Ni 4*p*-orbital degeneracy in this molecular geometry. Single-crystal X-ray diffraction and quantum chemical calculations both reveal a toroidal band of electron density perpendicular to the *C*_3_ axis of the complex, which may be attributed to delocalized, multicenter aromatic NiBe_6_ bonding.

## Introduction

In contrast to the heavier transition elements, the chemistry of the 3*d*-metals is dominated by lower valence states: di- and trivalent species are known for all ten elements, with the former prevailing later in the series.^1−4^ Known valence states for nickel span the range from zero- to tetra-valent (i.e., NiX_0_–NiX_4_), but the divalent (NiX_2_) state predominates under ambient conditions.^5,6^ Higher nickel valence states (NiX_3_ and NiX_4_) have been invoked for key intermediates in nickel-catalyzed carbon–carbon and carbon-heteroatom cross-couplings, and in carbon–hydrogen bond functionalization reactions.^7−10^ In Nature, access to the trivalent state of nickel is vital to the function of a variety of metalloenzymes, such as [NiFe] hydrogenases and nickel superoxide dismutase.^11,12^ Metal–ligand bonding in nickel complexes is typically weaker than that in palladium or platinum analogues.^13^ Additionally, successive ionization energies increase more rapidly for Ni than for Pd or Pt, meaning that the preparation of stable high-valent complexes is considerably more challenging for Ni than for the heavier elements of group 10.^13^ Indeed, only a few NiX_4_ complexes featuring nickel in the tetravalent state – the highest known valence for this element – have been isolated.^1,2^ Divalent 20-electron NiCp_2_ (and substituted derivatives thereof) can be oxidized to the corresponding dication and a handful of organometallic NiX_4_ complexes have been crystallographically characterized.^7−9,14−18^ However, although a small number of fluoride-ligated tetravalent nickel species have been isolated, (e.g., K_2_[NiF_6_] and NiF_4_) these are often unstable and no nickel analogue of PtF_6_ has been prepared.^19^

Here, some important concepts should be clarified for the reader. The International Union of Pure and Applied Chemistry (IUPAC) defines “valence” as “the maximum number of atoms that may combine with an atom of the element under consideration...”. In other words, valence is equal to the number of covalent bonds an element forms, or the number of X-type (one-electron) ligands bonded to that central element.^20,21^ “Coordination number” – purely the number of atoms in the primary coordination sphere of the central atom – is distinct from “valence” in this sense.^20^ The more nuanced concept of “oxidation state” is defined as “the atom’s charge after ionic approximation of its bonds.”.^21^ It relies upon the apportioning of electrons in a covalent bond to one of the partners, typically based on electronegativity arguments, and is known to be fraught with difficulty.^22^ Indeed, the “oxidation state” and “valence” of a metal ion may be inequivalent due to (i) the presence of homoelemental (or homopolar) bonds; or (ii) ligand non-innocence, which is a concept of particular relevance to high-valent metal complexes.^20,23^ Indeed, while known formal nickel oxidation states range from −2 to +4 (in the condensed phases), X-ray absorption spectroscopy (XAS) measurements and complementary quantum chemical calculations indicate that, in the vast majority of cases, this manifests as a physical d^8^ or d^9^ configuration, regardless of formal oxidation state.^5,6,24^ For example, tetravalent K_2_[NiF_6_] (which is a NiX_4_L_2_ complex, as a number of X-type ligands (fluoride) equal to the overall charge on the complex are converted to L-type ligands) has a physical nickel oxidation state of +1 (vide infra).^24^

The activation of H–H and B–B bonds by transition metals (yielding metal complexes featuring hydride [H]^−^ or boryl [BR_2_]^−^ ligands, respectively) has been widely studied both from the viewpoint of structure and bonding, and within the context of hydrogenation/borylation catalysis.^25,26^ We recently reported diberyllocene (CpBeBeCp; cyclopentadienyl anion (Cp)), a stable complex with a Be–Be bond.^27,28^ Given that beryllium is adjacent to boron in the Periodic Table, we envisaged that a Be–Be bond might similarly undergo addition reactions at transition metal centers, yielding two “beryllyl” [BeR]^−^ ligands.^29^ Considering the very low electronegativity of beryllium (1.57 on the Pauling scale; cf. boron, 2.04), we hypothesized that these X-type beryllyl ligands might be even more potently electron releasing than their boryl counterparts, which are known to be very strongly σ–donating.^30^

Here we report a stable, crystalline hexavalent nickel (NiX_6_) complex, Ni(BeCp)_6_ (**1**), formed by the reductive addition of three Be–Be bonds to zerovalent nickel. Crystallographic, spectroscopic, and quantum chemical studies suggest that a closed-shell *C*_3*v*_ structure is the ground state, with an alternative pseudo-octahedral geometry triplet state lying much higher in energy. Notably, due to the coordination of six BeCp metallo-ligands to the nickel center in **1**, this complex exhibits an inverted ligand field. Analysis of the DFT-derived molecular orbital picture also reveals a delocalized toroidal orbital composed of the in-phase combination of the Ni 4*s*-orbital with the six Be 2*s*-orbitals, which fulfils many criteria associated with aromaticity and may help to stabilize the observed *C*_3*v*_ molecular geometry. Remarkably, this toroidal feature is also detected experimentally in single crystal X-ray diffraction studies.

## Results and Discussion

### Synthetic and Spectroscopic Studies

**Figure 1:**
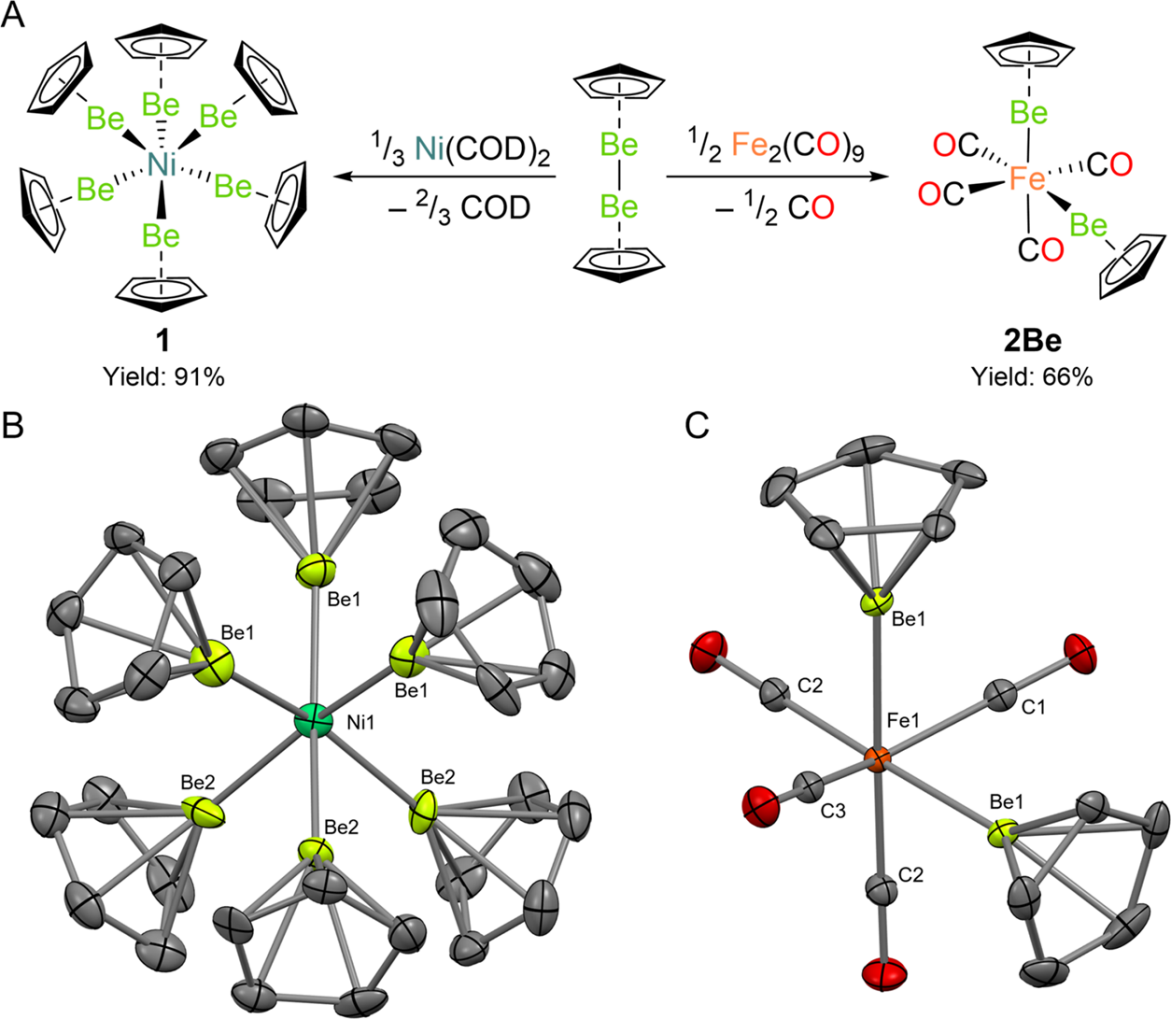
Syntheses and crystal structures of “beryllyl” complexes of nickel **(1)** and iron **(2Be)**. (A) Synthesis of **1** and **2Be** through addition of Be–Be bonds. (B) Molecular structure of **1** in the solid state, as determined by X-ray crystallography. (C) Molecular structure of **2Be** in the solid state, as determined by X-ray crystallography. Hydrogen atoms in B and C are omitted for clarity.

Reaction of two equivalents of diberyllocene with Fe_2_(CO)_9_ in benzene led to the formation of two equivalents of the 18-electron, diamagnetic bis(beryllyl) complex *cis*-Fe(BeCp)_2_(CO)_4_ (**2Be**), isolated in 66% yield (Figure 1A, right). Pale yellow crystals of **2Be** suitable quality for single-crystal X-ray diffraction (SC XRD) experiments could be obtained from concentrated hexane solutions (Figure 1C). In the solid state, **2Be** exhibits a distorted octahedral geometry featuring two mutually *cis*-BeCp groups. The carbonyl ligands *cis* to the beryllyl groups bend out of the axial plane toward the beryllium centers (C1–Fe1–C3 angle = 137.21(7)°), suggesting Fe has been (partially) reduced. Indeed, this distortion is more pronounced than that observed for the mutually *trans* carbonyl ligands in the isostructural iron-bis(boryl) complex *cis*-Fe(BCat*)_2_(CO)_4_ (**2B**; BCat* = BO_2_C_6_H_3_-4-^*t*^Bu; 166.0(4)°) and -bis(hydride) complex *cis*-Fe(H)_2_(CO)_4_ (**2H**; 148.5(1.5)°), indicating the even more potent σ-donor properties of the BeCp ligand.^31−33^ The Fe–Be distance in **2Be** (2.1884(14) Å) is consistent with the sum of the single bond covalent radii of the elements (2.18 Å).^34^ Notably, the Fe–C1 and −C3 distances, associated with the mutually *trans* carbonyl ligands (1.7625(17) and 1.7680(16) Å, respectively) are markedly shorter than the Fe–C2 distance (1.7886(11) Å) for the carbonyl ligands *trans* to BeCp. This geometric feature is also consistent with the strongly σ-electron donating properties of the beryllyl metallo-ligand.^32^

Complex **2Be** was probed by multinuclear nuclear magnetic resonance (NMR) spectroscopy. In the ^1^H NMR spectrum, a single resonance at 5.76 ppm is measured for the protons of the cyclopentadienyl ligands, i.e., in the region typical of beryllium-coordinated cyclopentadienyl ligands (cf. diberyllocene, 5.73 ppm).^27^ The ^9^Be NMR spectrum of **2Be** consists of a single resonance at −18.0 ppm, which is shifted markedly downfield compared with that measured for diberyllocene (−27.6 ppm). ^9^Be NMR shifts are sensitive to the electron density at a given beryllium center, particularly in the case of CpBeX species.^35^ The low-field shift for **2Be** is indicative that the two X-type BeCp ligands are discrete and are not connected by a Be–Be bond. The ^13^C{^1^H} NMR spectrum of **2Be** consists of a resonance at 105.8 ppm, corresponding to the carbon atoms of the Cp ligands, as well as resonances at 211.4 and 212.1 ppm, associated with the carbonyl ligands. These resonances for **2Be** are shifted downfield compared to those associated with the carbonyl ligands in the bis(boryl) analogue **2B** (204.3 and 202.2 ppm).^32^

To further scrutinize the electronic properties of the beryllyl ligand, attenuated total reflection infrared (ATR IR) measurements were carried out on **2Be**. The stretching frequencies of ancillary carbonyl ligands are a widely employed probe of the electron-donating properties of the other ligands coordinated to the same metal center. Four IR-active carbonyl stretching bands for **2Be** are measured at 2010, 1983, 1887, 1870 cm^–1^, consistent both with local *C*_2*v*_ symmetry and with the pattern of bands in the simulated IR spectrum of **2Be** (Figure S8). Critically, these carbonyl stretches are measured at much lower frequencies than those of isostructural complexes such as **2B** (2117, 2050, 2036, 2000 cm^–1^) and *cis*-Fe(SiMe_3_)_2_(CO)_4_ (**2Si**; 2069, 2006, 2000, 1979 cm^–1^).^32,36^ These data provide further support for the hypothesis that BeCp is an extremely strongly σ-electron releasing ligand, showing it to be even more potent than boryl and silyl donors.

Having established the unprecedented σ-donor capabilities of BeCp as a covalently bound X-type ligand, we sought to exploit these electron-releasing capabilities in the synthesis of 3*d*-metal complexes featuring hitherto unknown (high) valence states. Hence, we investigated the reaction of diberyllocene with bis(cyclooctadiene)nickel(0) (Figure 1A). Analysis of the benzene reaction mixture by multinuclear NMR spectroscopy indicated that three equivalents of the beryllium starting material had been consumed and that free cyclooctadiene was generated. Colorless needle-like crystals formed readily at room temperature, which SC XRD revealed to be the 16-electron, NiX_6_ complex Ni(BeCp)_6_ (**1**; Figure 1B), isolated in 91% yield. Access to the hexavalent state of Ni in **1** can be attributed to the electronic (and steric) properties of the beryllyl ligands.^37−39^ Again, we note that synchrotron studies indicate that an element’s valence state (i.e., the number of covalent bonds/X-type ligands) and its oxidation state (which apportions charge within those bonds) are often inequivalent for high-valent metal complexes.^24^ Nonetheless, complex **1** is unambiguously a NiX_6_ system (i.e., CpBe is an X-type ligand), regardless of the oxidation state of the central nickel atom. This contrasts with precedented dianionic [NiF_6_]^2–^ complexes, for example, which are tetravalent NiX_4_L_2_ systems (vide infra).^20−22^

Homoleptic complex **1** exhibits a *C*_3*v*_ geometry, featuring a six-coordinate Ni center ligated exclusively by BeCp metallo-ligands. When viewed perpendicular to the *C*_*3*_ axis (Figure 1B), there are two “decks” of three Be atoms, one with narrower Be1–Ni1–Be1 angles (81.6(3)°), and one with wider Be2–Ni1–Be2 angles (101.9(2)°). Within **1**, the Ni1–Be1 and Ni1–Be2 distances are 2.090(8) and 2.117(13) Å, respectively, which are statistically indistinguishable by the 3σ-criterion, and comparable to the sum of the covalent radii for these elements (2.12 Å). Crucially, all Be···Be distances are >2.7 Å, which is significantly greater than (i) the sum of the single-bond covalent radii for beryllium (2.04 Å);^34^ (ii) the Be–Be bond in diberyllocene (2.0545(18) Å);^27^ and even (iii) the Be–Be distance in diatomic Be_2_ (2.45 Å), which features a Be–Be bond order of zero.^40^ These separations also approach the sum of the van der Waals radii for Be (3.06 Å).^41^ Therefore, these data imply that **1** does not feature any Be–Be bonding, a point further evidenced by NMR spectroscopy and quantum chemical calculations carried out on **1** (vide infra).

Structurally, complex **1** bares superficial resemblance to Pt(ZnCp*)_6_ (**3**; pentamethylcyclopentadienyl anion (Cp*)). Complex **3** exhibits local *D*_2*h*_ geometry at platinum and six one-electron X-type ZnX ligands (which are isolobal with BeCp) covalently bonded to the central metal atom.^42^ Additionally, considering that the nickel center in **1** is ligated entirely by X-type metallo-ligands, comparisons with the multimetallic clusters {[(Cp*)Ni](*μ*-ZnMe)_4_[Ni(ZnCp*)_2_(ZnMe)_2_]} (**4**) and {[(C_6_H_5_Me)Ni](*μ*-ZnMe)_4_[Ni(ZnCp*)_2_(ZnMe)_2_]} (**4′**), can also be drawn.^43^ In this context, four-coordinate [Ni(AlCp*)_4_]^+^, which is a monovalent NiL_4_X complex bearing four two-electron L-type AlX metallo-ligands, is also worthy of mention.^44^

The ^1^H NMR spectrum of **1** features a single resonance at 5.71 ppm, corresponding to the protons of the Cp ligands, which is comparable to the ^1^H NMR shift of **2Be** (5.76 ppm).^27^ The ^9^Be NMR spectrum for **1** features a single resonance at −16.7 ppm, which is the most downfield of any reported CpBeX complex.^35^ Aromatic ring currents may explain the deshielding of the six beryllium centers in **1** (vide infra). Notably, as discussed for **2Be**, this chemical shift may also evidence the absence of Be–Be bonding in **1**, which is known to lead to high electron density at Be and high-field ^9^Be NMR chemical shifts (cf., diberyllocene, –27.6 ppm).^27^ Complex **1** exhibits a single resonance at 105.7 ppm in its ^13^C{^1^H} NMR spectrum, associated with the Cp carbon atoms, which is near-identical to the corresponding signal in the ^13^C{^1^H} NMR spectrum of **2Be** (105.8 ppm).

Complex **1** was also investigated by vibrational spectroscopy. Both the ATR IR and Raman spectra collected for **1** closely match the simulated spectra for this complex (Figures S9 and S10; vide infra). While the IR spectrum of **1** is relatively uninformative, the Raman spectrum features broad inelastic scattering bands centered at 107 and 577 cm^–1^, which are calculated to correspond to an *A*_1_-symmetry NiBe_6_ breathing mode and *E*-symmetry Ni–Be stretching modes, respectively.

### Computational Studies

**Figure 2:**
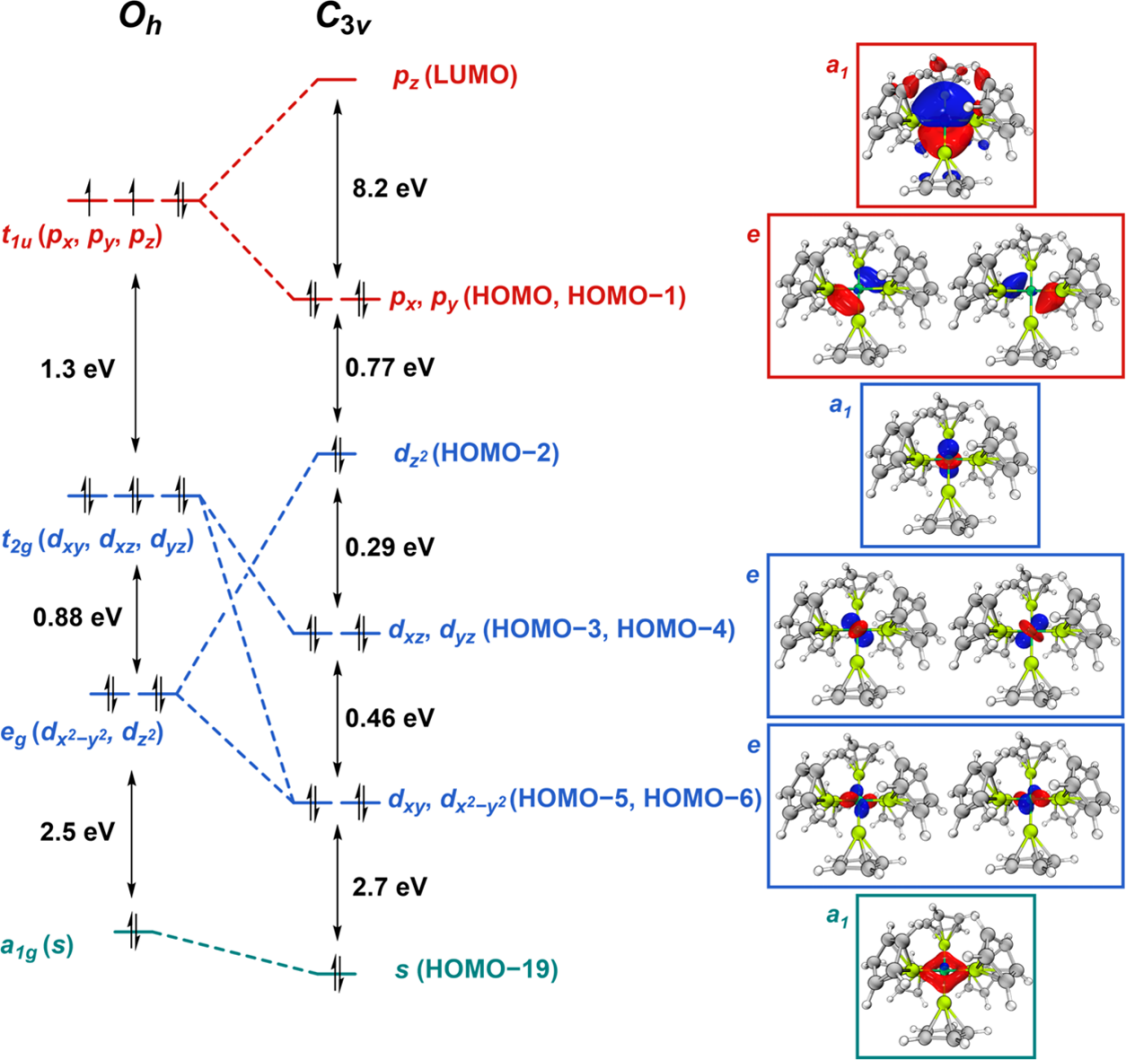
(Left) Relative molecular orbital scheme for **1′** (*O*_*h*_) and **1** (*C*_3*v*_); (Right) corresponding molecular orbitals (0.075 a.u) calculated for **1**.

Quantum chemical calculations were employed in order to gain a deeper understanding of the electronic structure and geometry of **1**. The geometry of the complex was optimized at the ωB97X-D4 DEF2-QZVPP level, yielding a complex with *C*_3*v*_ symmetry, which closely resembles the crystallographically determined parameters of **1**. The structure of Ni(BeCp)_6_ with the NiBe_6_ core constrained in *O*_*h*_ geometry (**1′**) was also optimized, showing the complex to be significantly higher in energy than **1** (Δ*E* = +46.0 kcal mol^–1^). Within homoleptic **1**, Ni (Pauling electronegativity, 1.91) is ligated exclusively by Be-centered donors (Pauling electronegativity, 1.57); and hence, has an inverted ligand field (Figure 2).^23^ As predicted by Hoffmann, in this regime high-lying orbitals should have a large degree of ligand character, whereas lower energy orbitals are mainly metal centered.^23^ As such, all Ni *d*-orbitals in **1** are fully occupied, and their ordering is the inverse of that expected from a “classical” transition metal complex. In addition, the lowest unoccupied molecular orbital (LUMO), highest occupied molecular orbital (HOMO), and HOMO–1 comprise large contributions from beryllium (vide infra). The electronic structure of **1**, therefore, supports the hypothesis that powerfully σ-electron releasing metallo-ligands can induce this electronic structuring.^23^ Interestingly, there have been previous suggestions that many NiX_4_ complexes feature an inverted ligand field, which has been computationally demonstrated to influence their reactivity in C–C bond-forming reactions.^7,24,45^

While **1** is unambiguously a hexavalent NiX_6_ species, we also wished to address the question of nickel oxidation state in this complex. X-ray absorption spectroscopy (XAS) is widely regarded as the most appropriate technique for this purpose, as shown explicitly for nickel complexes.^24^ However, the instability of complex **1** under vacuum makes such measurements very challenging: the complex eliminates diberyllocene under active vacuum (<10^–2^ mbar) to generate metallic nickel. It has recently been shown by Lancaster and co-workers that the nickel valence electron count determined experimentally by Ni L_2,3_-edge XAS correlates very well with that derived from Löwdin population analysis (LPA) calculations.^24^ Therefore, we used this specific computational technique to assess the oxidation state of nickel in **1**. As aforementioned, the nickel 3*d*-orbitals of **1** are filled, as is also evidenced by the LPA analysis (9.8 e^–^). Furthermore, LPA indicates that the nickel 4*s*- and 4*p*-orbitals are partially occupied (1.0 and 0.2 e^–^, respectively). On this basis, a physical nickel oxidation state between 0 and −1 could be tentatively assigned for **1**. Thus, this complex would appear to represent a remarkable example of a high-valent, low-oxidation state nickel species.^23^ By means of comparison, the tetravalent NiX_4_L_2_ species [NiF_6_]^2–^ has been shown by LPA to possess a 3d^8^4s^0.5^4p^0.5^ electronic configuration, implying a physical oxidation state of +1 for nickel. This contrasts starkly with the formal nickel(IV) formulation for [NiF_6_]^2–^, which would correspond to a 3d^6^ configuration.^24^ Thus, as with many high-valent metal complexes, it would seem that the oxidation state and valence state of the nickel center in **1** are inequivalent.

To better understand the electronic structure of **1**, we undertook a relative comparison of the frontier orbitals of this complex and hypothetical octahedral complex **1′** (Figure 2). Projecting the *z*-axis parallel to the molecular *C*_*3*_ axis, calculations on **1** indicated that the highest energy nickel-based valence d-orbital is the nonbonding *d*_*z*^2^_ orbital (HOMO–2). The next highest energy nickel *d*-orbitals are the pseudodegenerate *d*_*xz*_ and *d*_*yz*_ (HOMO–3 and HOMO–4, respectively), which, due to their alignment along the *z*-direction, are also not able to overlap effectively with Be-based orbitals. The lowest energy Ni *d*-orbitals are the pseudodegenerate *d*_*x*^2^–*y*^2^_ and *d*_*xy*_ orbitals (HOMO–5 and HOMO–6, respectively), which engage in constructive overlap with the 2*s*-orbitals of four and two Be centers, respectively. The higher energy Ni 4*p*_*x*_ and 4*p*_*y*_ orbitals (HOMO and HOMO–1) are pseudodegenerate, with the former overlapping constructively with two pairs of Be 2*s*-orbitals and the latter with all six Be 2*s*-orbitals. The high ligand character of these orbitals is consistent with an inverted ligand field (Table S7).^23^ The LUMO of **1** corresponds to the Ni 4*p*_*z*_ orbital (also high in Be 2*s* character; Table S7), which lies much higher in energy (HOMO–LUMO energy gap = 8.2 eV). Hence, for **1** we postulate that the stabilization of the *p*_*x*_*-* and *p*_*y*_-orbitals, and destabilization of *p*_*z*_-orbital (enabled by descent to *C*_3*v*_ symmetry), plays a significant role in determining the (diamagnetic) singlet ground state of 16-electron complex **1**. The nickel 4*s*-orbital is also engaged in a significant bonding interaction with the beryllyl ligands, contributing to the HOMO–19. Strikingly, this delocalized molecular orbital is toroidal in shape, occupying the “equatorial” region between the two “decks” of three beryllium atoms when viewed perpendicular to the *C*_3_ axis. This orbital is composed of the in-phase combination of the Ni 4*s*-orbital with the six Be 2*s*-orbitals. In contrast to closed shell complex **1**, hypothetical complex **1′** is a triplet with two unpaired electrons in the three degenerate Ni 4*p*-orbitals (*t*_1*u*_ set). Due to the inverted ligand field, the triply degenerate *t*_2*g*_ set (*d*_*xy*_, *d*_*xz*_ and *d*_*yz*_) are the highest energy Ni valence *d*-orbitals and the doubly degenerate *e_g_* set (*d*_*z*^2^_ and *d*_*x*^2^–*y*^2^_) are the lowest energy Ni *d*-orbitals (Figures 2 and S16).^23^

Comparisons of the orbital manifold of **1** with [NiF_6_]^2–^ – formally a high-valent, high-oxidation state nickel complex – can also be made. First, [NiF_6_]^2–^ also features an inverted ligand field.^24^ Indeed, the nickel (3*d*-orbital) character of the occupied *t*_2*g*_ set is only 40%, with significant contributions coming from the fluoride lone-pairs.^24^ Furthermore, the nickel parentage of the occupied *a*_1*g*_ and *t*_1*u*_ sets is very low, at 7% (4*s*-orbital) and 3% (4*p*-orbitals), respectively.^24^ This mirrors the high degree of ligand character calculated for the analogous orbital sets of **1** (Table S7). Thus, in spite of superficial differences and the contrasting natures of the ligand sets of **1** and [NiF_6_]^2–^, the electronic structures of these two complexes show remarkable similarities. In the case of [NiF_6_]^2–^, the inverted ligand field can be ascribed to the similar energies of the fluorine 2*p*- and the nickel 3*d*-orbitals, given the oxidized nature of the nickel center.^24^ In the case of **1**, ligand field inversion reflects the high energies of both the beryllium and the nickel frontier orbitals, stemming from the electron-rich nature of the nickel center.^23^ Indeed, in both of these high-valent nickel complexes, the isoenergetic nature of the metal and ligand valence orbitals results in ligand non-innocence.^23^

Quantum theory of atoms in molecules (QTAIM) calculations were employed to examine the Ni–Be bonding and charge distribution within **1** (Figure S23 and Table S2). Bond paths (BPs) and bond critical points (BCPs) are located for all of the Ni–Be interactions. Topological parameters (Be–Ni ρ_bcp_ = 0.061 e^–^ Bohr^–3^; ∇^2^ρ_bcp_ = −0.014 e^–^ Bohr^–5^) are typical of Be–M interactions (e.g., Be–In, ρ_bcp_ = 0.055 e^–^ Bohr^–3^; ∇^2^ρ_bcp_ = −0.021 e^–^ Bohr^–5^) and indicative that the Be–Ni bonding in **1** is weakly covalent in nature.^46,47^ Deconvolution of the Ni–Be BCPs reveals that the principal contributing orbitals to these interactions are the HOMO–19 in combination with the HOMO–5/HOMO–6. Decisively, no BPs or BCPs corresponding to Be···Be interactions are located by these calculations. This finding provides further evidence for the hexavalent nature of the Ni center in **1**, which is bonded to six discrete X-type Be ligands, and not to L-type Be–Be σ-bonds.^13^ QTAIM basin analysis of **1** returns charges of −3.98 for the Ni center and +1.50 for all Be atoms, indicating that the Ni atom is extremely charge rich due to the coordination of the powerfully σ-electron-releasing BeCp ligands. QTAIM calculations also detect a network of BCPs between the hydrogen atoms of Cp ligands. Hence, the fact that **1** can even be isolated may arise, in part, from the stabilizing dispersion interactions between Cp ligands, in addition to the steric shielding these organic groups afford the [NiBe_6_]^6+^ core.^48^

**Figure 3:**
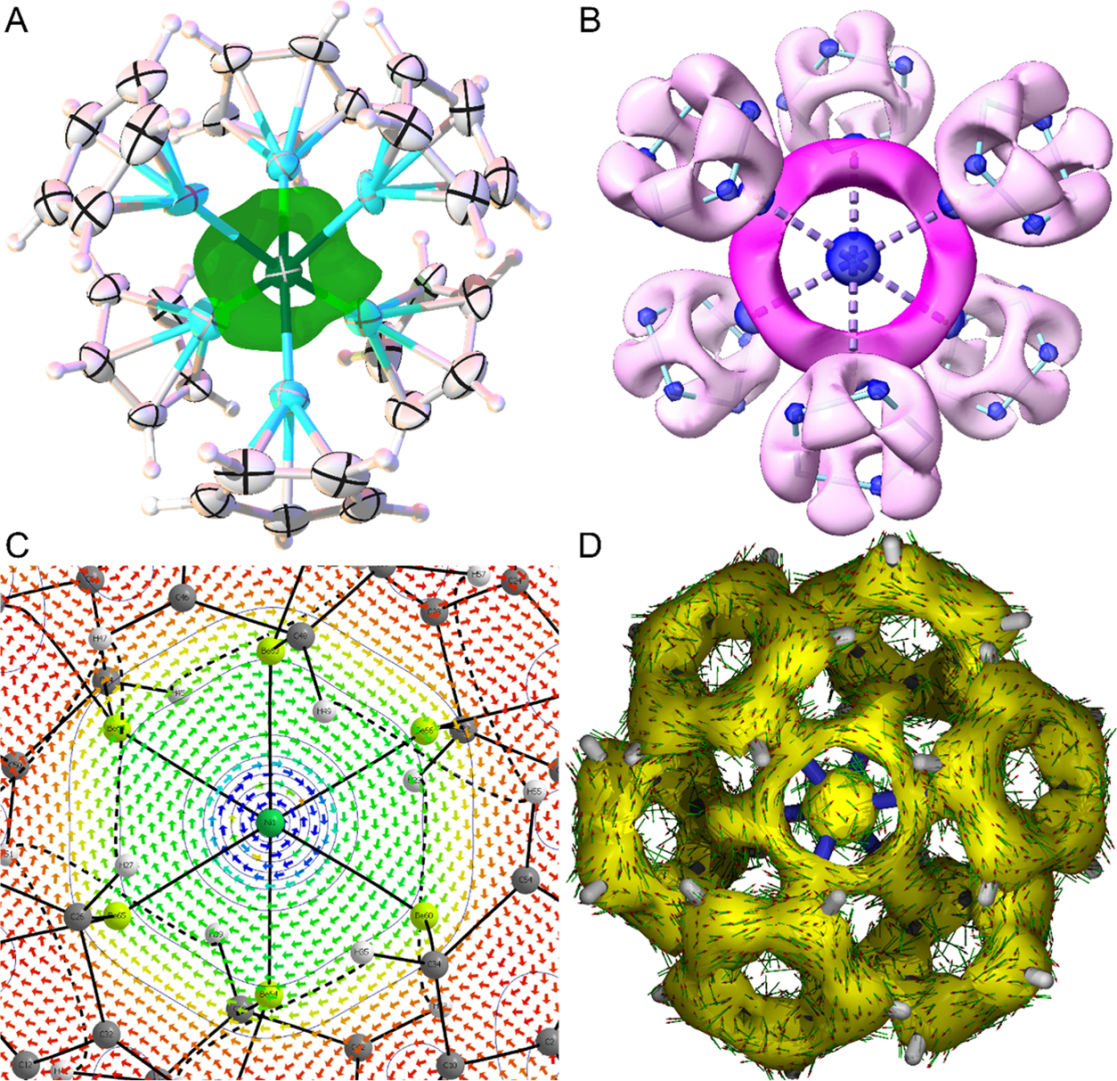
(A) SC XRD residual electron density map for **1**, with green ring representing an area of residual density; (B) ELF isosurface for **1** (hydrogen atoms omitted for clarity; 0.7 au); (C) Profile of current density within **1** in the plane of the NiBe_6_ toroidal HOMO–19. The blue to red color scale indicates strong (*J*(r) ≥ 0.001 au) to weak currents (*J*(r) = 0.0000). A clockwise (diamagnetic) current can be seen; (D) ACID plot for **1** at 0.05 au isosurface value. The clockwise direction of the induced current (illustrated by red arrow heads) indicates aromatic delocalization.

The electron localization function (ELF) was calculated for **1**. Inspection of the isosurface reveals a delocalized, toroidal region of electron density, which occupies a space between the two “decks” of three beryllium atoms, perpendicular to the *C*_3_ axis–thus, resembling the HOMO–19 of **1** in both its position and profile (Figure 3B). This delocalized bonding is reflected in the multicenter bond order calculated for the NiBe_6_ moiety of **1** (0.023; cf., that for benzene 2.8 × 10^–4^). Additionally, Electron Density of Delocalized Bonds (EDDB) calculations indicate that, aside from the 36 delocalized electrons associated with the Cp ligands, approximately two electrons are completely delocalized across the NiBe_6_ unit.^49^

Given the delocalized nature of the NiBe_6_ bonding in **1**, we performed nucleus-independent chemical shift (NICS) calculations on the complex.^50^ As the Ni center occupies the site at the center of the ring in **1**, NICS(0) (the negative of the isotropic magnetic shielding at the center of the ring) is uninformative. However, the NICS(1) value (the negative of the isotropic magnetic shielding 1 Å perpendicularly above and below the toroidal ring center; −17.6 ppm) for **1** is consistent with an aromatic system (NICS(1) for benzene = −10.0 ppm). Aromaticity in **1** is also evidenced by inspection of the NICS_*zz*_ scan–the variation of the out-of-plane *zz* component of the shielding tensor (Figure S36). In the region between +0.5 and +2.5 (and −0.5 and −2.5), NICS_*zz*_ values are negative and decay rapidly with respect to the perpendicular distance from the ring centroid, which is also indicative of aromaticity.^50^

Ring current analysis was also performed on **1**.^51^ This reveals **1** to feature a substantial diamagnetic ring current in the region associated with the delocalized seven-center two-electron bond (Figure 3C). Around the inner rim of this ring, directly encircling the Ni center, a paramagnetic ring current is observed. Both features are mirrored in benzene and are indicative of aromaticity in **1**.^50,51^ Similarly, QTAIM magnetizability analysis was performed on **1**. The out-of-plane component of the atomic and bond magnetizability (χ_*zz*_^Atom^ and χ_*zz*_^(*X*|*Y*)^, respectively) are useful probes for ring currents. In the case of **1**, χ_*zz*_^Atom^ Ni is −46.0 ppm, and average χ_*zz*_^(Be|Ni)^ is −2.4 ppm. For reference, χ_*zz*_^Atom^ for the C atoms of benzene is −3.4 ppm and χ_*zz*_^(C|C)^ is −5.1 ppm. Again, this further evidences the diamagnetic currents at Ni and Be and the possible aromaticity of **1**.^51^

The anisotropy of induced current density (ACID) was also used to assess the aromaticity of **1**.^52^ The ACID plot represents a visualization of the density and the direction of the ring current induced when an external magnetic field is applied perpendicularly to the delocalized bonding system (Figure 3D). The direction of the current density vectors (clockwise) is the same for the (aromatic) Cp ligands and the delocalized NiBe_6_ bonding, which provides further evidence for aromaticity in **1**.^52^ Aromatic deshielding may explain the downfield shift of the ^9^Be NMR resonance observed for **1**. Remarkably, there is also experimental evidence for this calculated electronic feature. The residual electron density map, derived from SC XRD measurements on **1** (refined using nonspherical atomic form factors) at a range of temperatures, shows a clear toroidal band of electron density in the same region that the ELF isosurface is calculated to occupy (Figure 3A).^28,53,54^

## Conclusions

In summary, new transition metal beryllyl complexes − Ni(BeCp)_6_ (**1**) and *cis*-Fe(BeCp)_2_(CO)_4_ (**2Be**) – have been synthesized. Study of **2Be** reveals the potent σ-donating character of X-type beryllyl ligands. This ligand property is crucial to the isolation of hexavalent nickel (NiX_6_) complex **1**, which is prepared by insertion of zerovalent nickel into three Be–Be bonds. Our calculations indicate that **1** features an inverted ligand field, which has been established for other high valent nickel complexes. Furthermore, we postulate that complex **1** adopts *C*_3*v*_ (rather than *O_h_*) geometry as this yields a large HOMO–LUMO gap, through the splitting of the *t*_1*u*_ (Ni 4*p*) orbitals. Quantum chemical analysis also indicates that the complex features seven-center two-electron delocalized bonding across the NiBe_6_ core, which may be aromatic in nature. Our work allows for comparisons of the addition chemistries of the H–H, B–B, and Be–Be bonds at low-oxidation state metal centers.

## Supporting Information Available

Materials and methods; experimental procedures; spectroscopic data; crystallographic details; and quantum chemical data (PDF)
