# Supplementary material for: A Crystalline NiX6 Complex
Source: J Am Chem Soc. 2024 Dec 13;146(51):35208–15. doi: 10.1021/jacs.4c12125 (PMC11673578; doi:10.1021/jacs.4c12125)
Supplement: Supplementary file 1 — ja4c12125_si_001.pdf [file ja4c12125_si_001.pdf]

Supporting Information

A Crystalline NiX_6 Complex

Josef T. Boronski[1,2]*, Agamemnon E. Crumpton[1], Simon Aldridge[1]*

1) Chemistry Research Laboratory, Department of Chemistry, Oxford, OX1 3TA, U.K.

2) Department of Chemistry, Molecular Sciences Research Hub, Imperial College London, 82 Wood Lane, White City, London, UK

Experimental and Methods

Contents

General Experimental Considerations – S2

Synthesis of Novel Compound – S2

Spectroscopic Data – S4

Crystallographic Data – S10

Computational Details – S12

Molecular Orbitals – S12

QTAIM – S18

ELF and LOL – S21

NBO – S23

AdNDP – S24

EDDB – S27

NICS – S28

Ring Current Analysis and Magnetizability – S30

ACID – S33

References – S35

General Experimental Considerations:

All manipulations were carried out using Schlenk line or glovebox techniques under an atmosphere of argon or dinitrogen. Solvents were dried by passage through activated alumina towers, dried with NaK_2 and degassed before use. Solvents were stored over NaK_2. NMR spectra were measured in C_6D_6 which was dried over NaK_2, with the solvent being distilled under reduced pressure, degassed by three freeze-pump-thaw-cycles and stored under argon in a Teflon valve ampoule. NMR samples were prepared under argon in 5 mm Wilmad 507-PP tubes fitted with J. Young Teflon valves. NMR spectra were measured on a Bruker Avance III HD Nanobay 400 MHz NMR spectrometer equipped with a 9.4 T magnet or a Bruker Avance III NMR 500 MHz NMR spectrometer equipped with a 11.75 T magnet and a ^{13}C detect cryoprobe. ^{1}H and $^{13}C\{^{1}H\}$ NMR spectra were referenced internally to residual protio-solvent (^{1}H) or solvent (^{13}C) resonances and are reported relative to tetramethylsilane (δ = 0 ppm). ^{9}Be NMR spectra were referenced to a 0.43 M solution of $BeSO_4{\cdot}4H_2O$ in D_2O (δ = 0 ppm). Chemical shifts are quoted in δ (ppm) and coupling constants in Hz. FTIR spectra were recorded on a Bruker Alpha spectrometer with Platinum-ATR module. Elemental analyses were carried out by London Metropolitan University. Diberyllocene was prepared as described previously [1]. Transition metal precursors $Fe_2(CO)_9$ and $Ni(COD)_2$ were used as received.

Health warning: beryllium and its compounds are extremely toxic and can cause irreversible health effects through inhalation or skin contact. The work with beryllium-containing materials described herein was carried out by trained operator(s), with strict adherence to local and national rules/regulations [2].

Synthesis of Novel Compounds:

Synthesis of cis-Fe(BeCp)$_2$(CO)$_4$ (2Be): To a Schlenk flask equipped with a glass-coated stirrer bar was added a solid mixture of diberyllocene (10.0 mg, 0.067 mmol) and $Fe_2(CO)_9$ (12.3 mg, 0.034 mmol, 0.5 equiv.). Benzene (1 mL) was condensed into the vessel *in vacuo* at –196 °C. The orange solution was allowed to warm to room temperature and stirred for 96 hours. Solvent was removed from the solution *in vacuo*, yielding a brown solid. Soluble material was extracted with hexane (2 x 2 mL) and the solution was filtered. The pale orange solution was transferred to a λ-crystallization tube (fitted with a J. Young PTFE valve), frozen, and placed under a static vacuum. Slow concentration of the solution over the course of 16 hours led to the formation of a crop of pale yellow rod-like crystals of **2Be**, which were dried *in vacuo*. Yield: 14 mg, 66%. Single crystals of **2Be** suitable for X-ray diffraction experiments were obtained by slow concentration of a hexane solution in a λ-crystallization tube. Anal. Calcd for $C_{14}H_{10}Be_2FeO_4$: C, 53.20; H, 3.19. Found: C, 53.11; H, 3.09. ^{1}H NMR (400 MHz, C_6D_6, 298 K): δ = 5.76 (s, 10H, C_5H_5); ^{9}Be NMR (42 MHz, C_6D_6): δ = –18.0 ($w_{1/2}$ = 7.5 Hz); $^{13}C\{^{1}H\}$ NMR (101 MHz, C_6D_6): δ = 105.8 (C_5H_5), 211.4 (*C*O), 212.1 (*C*O). ATR-IR υ/cm^{-1}: 626 (s), 696 (w), 794 (m), 855 (m), 938 (m), 1014 (w), 1254 (w), 1870 (s), 1887 (m), 1983 (m), 2010 (m). Melting point: 476 K (*decomp.*).

Synthesis of Ni(BeCp)$_6$ (1): To a Schlenk flask equipped with a glass-coated stirrer bar was added a solid mixture of diberyllocene (15.0 mg, 0.10 mmol) and Ni(COD)$_2$ (9.3 mg, 0.034 mmol, 0.33 equiv.). Benzene (1 mL) was condensed into the vessel *in vacuo* at –196 °C. The yellow solution was allowed to warm to room temperature and stirred for 10 minutes. The solution was then allowed to stand at room temperature for 5 hours, leading to the formation of a large crop of colourless needle-like crystals. The mother liquor was decanted by filtration, the crystalline material was washed with pentane (3 x 2 mL), and dried briefly *in vacuo*. Yield: 15 mg, 91%. Single crystals of **1** suitable for X-ray diffraction experiments were obtained by allowing a benzene solution (0.5 mL) of diberyllocene (15.0 mg, 0.10 mmol) and Ni(COD)$_2$ (9.3 mg, 0.034 mmol, 0.33 equiv.) to stand undisturbed for 16 hours. Compound **1** decomposes under high vacuum and when heated. Anal. Calcd for $C_{30}H_{30}Be_6Ni$: C, 71.59; H, 6.01. Found: C, 71.55; H, 5.92. ^{1}H NMR (400 MHz, C_6D_6, 298 K): δ = 5.71 (s, 30H, C_5H_5); ^{9}Be NMR (42 MHz, C_6D_6): δ = –16.7 ($w_{1/2}$ = 25.1 Hz); ^{13}C{^{1}H} NMR (101 MHz, C_6D_6): δ = 105.7. Melting point: 326 K (*decomp.*).

Spectroscopic Data:

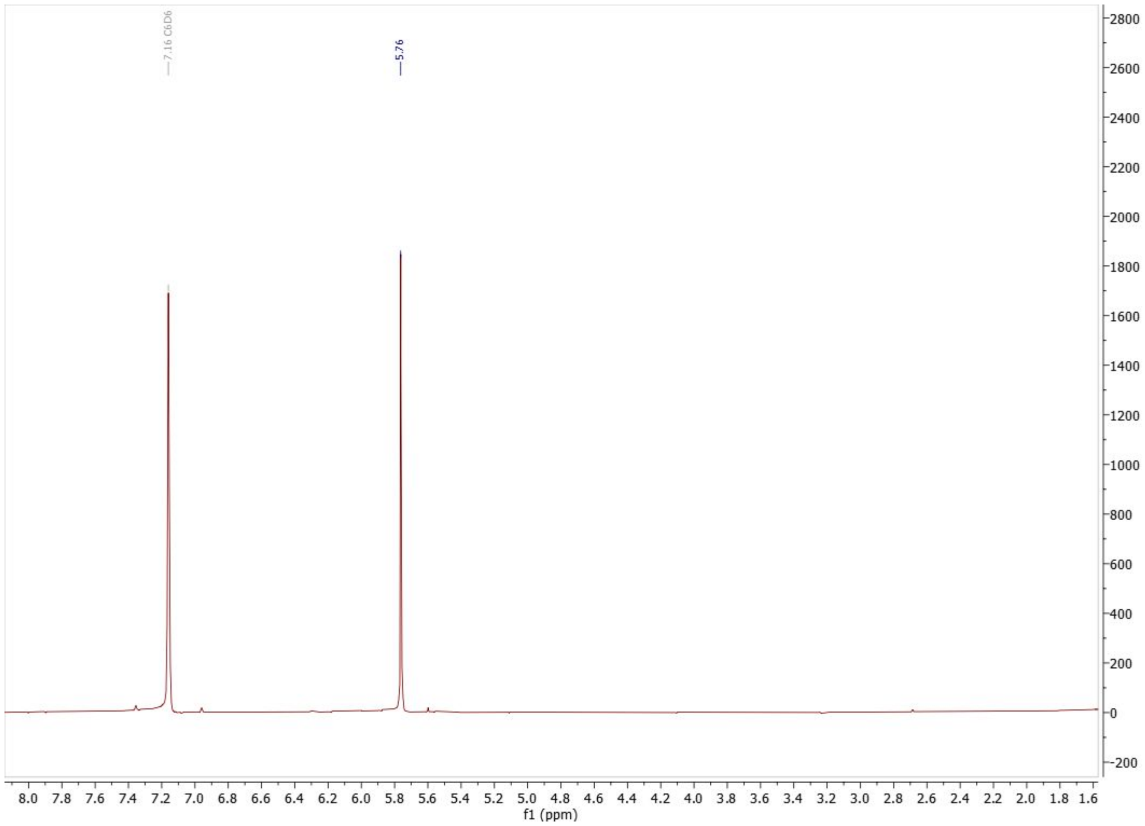

Figure S1: ^{1}H NMR spectrum of **2Be** in d_6-benzene.

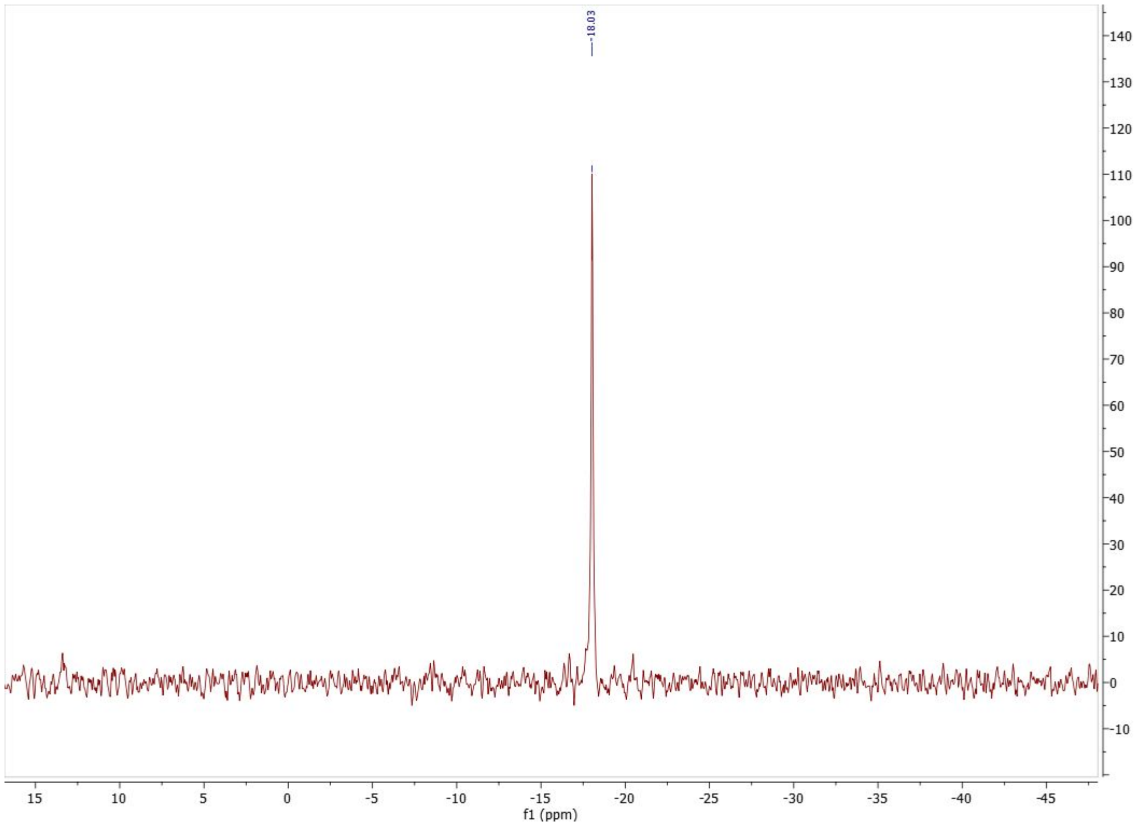

Figure S2: ^{9}Be NMR spectrum of **2Be** in d_6-benzene.

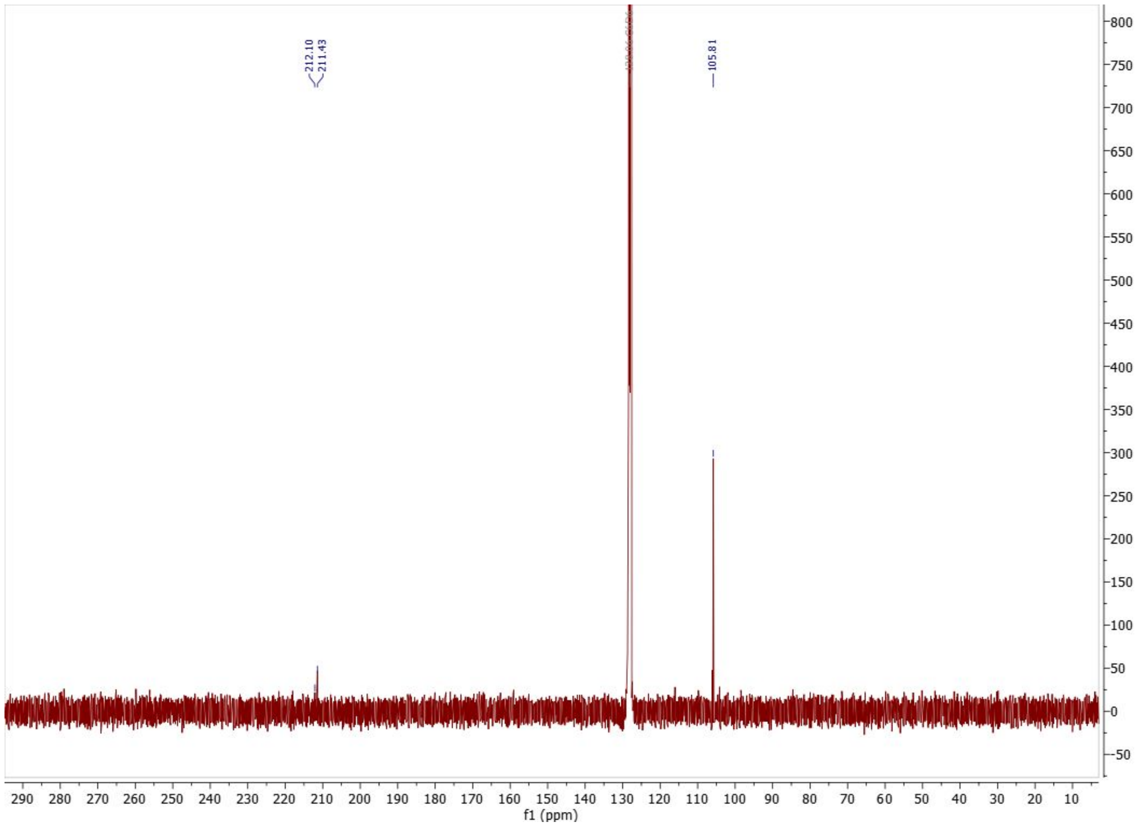

Figure S3: $^{13}C\{^{1}H\}$ NMR spectrum of **2Be** in d_6-benzene.

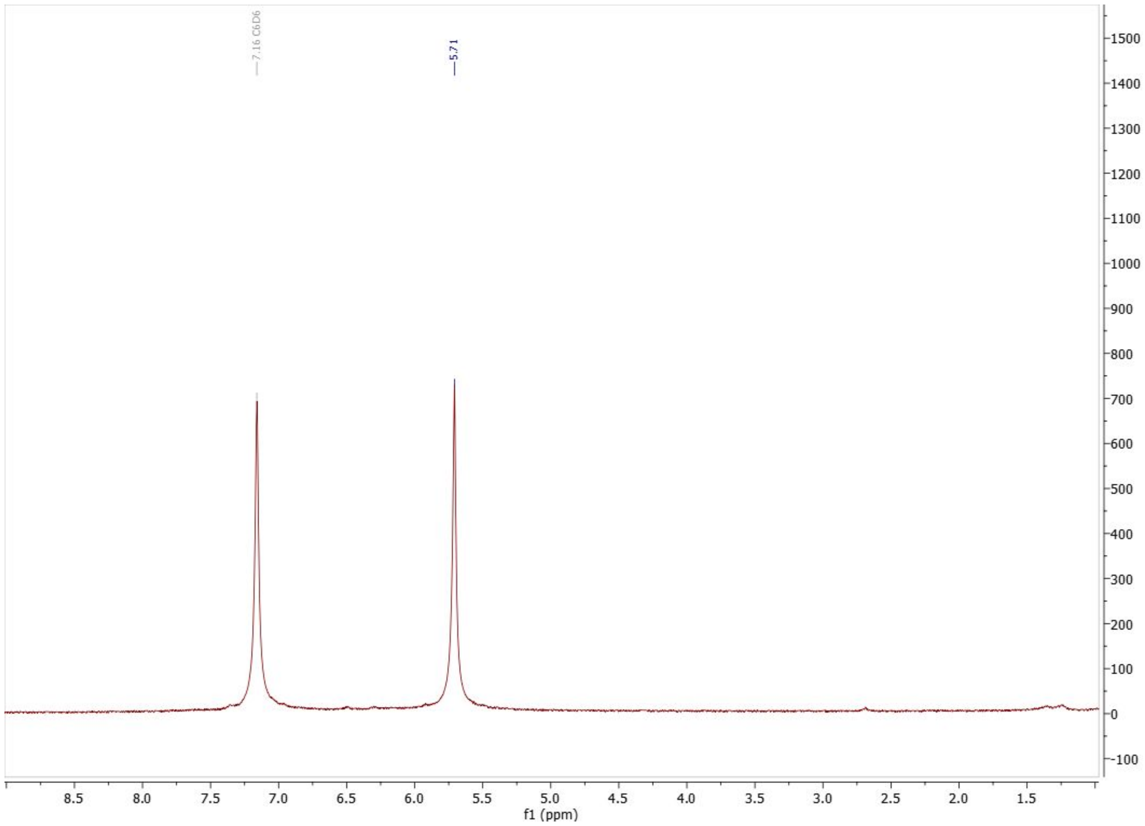

Figure S4: ^{1}H NMR spectrum of **1** in d_6-benzene.

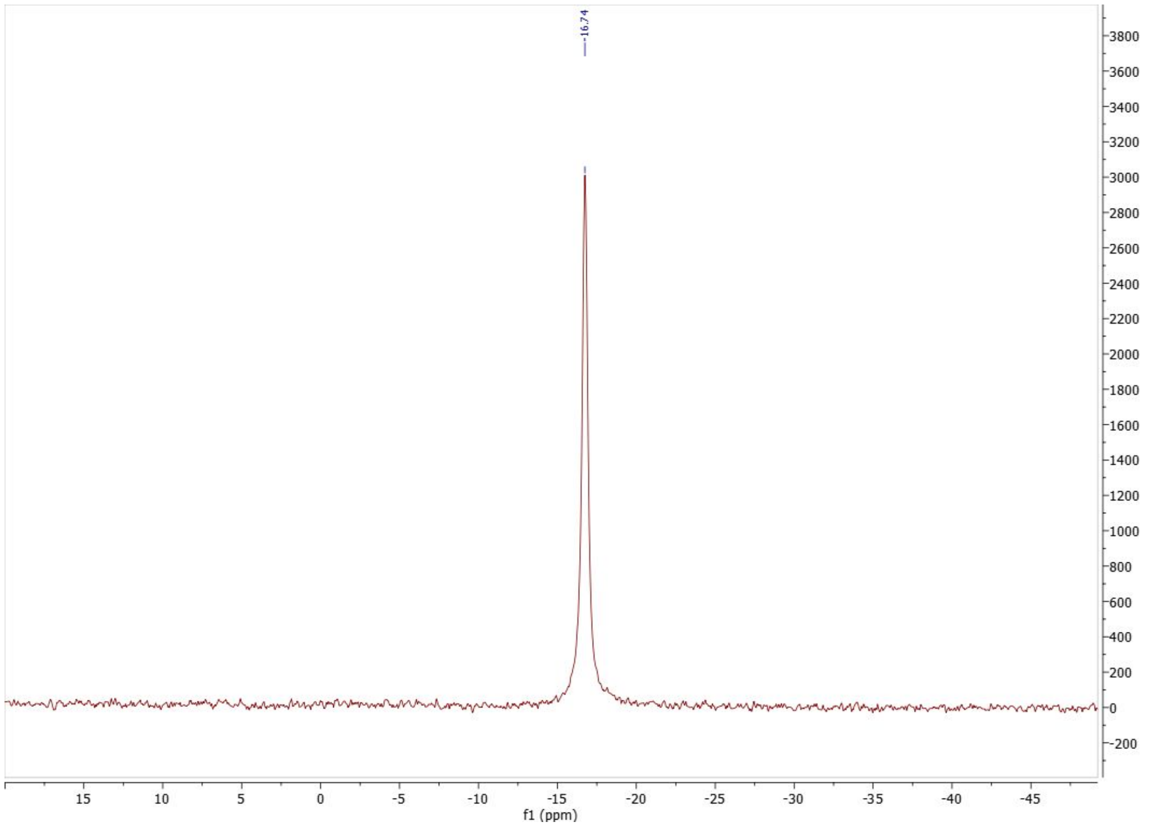

Figure S5: ^{9}Be NMR spectrum of **1** in d_6-benzene.

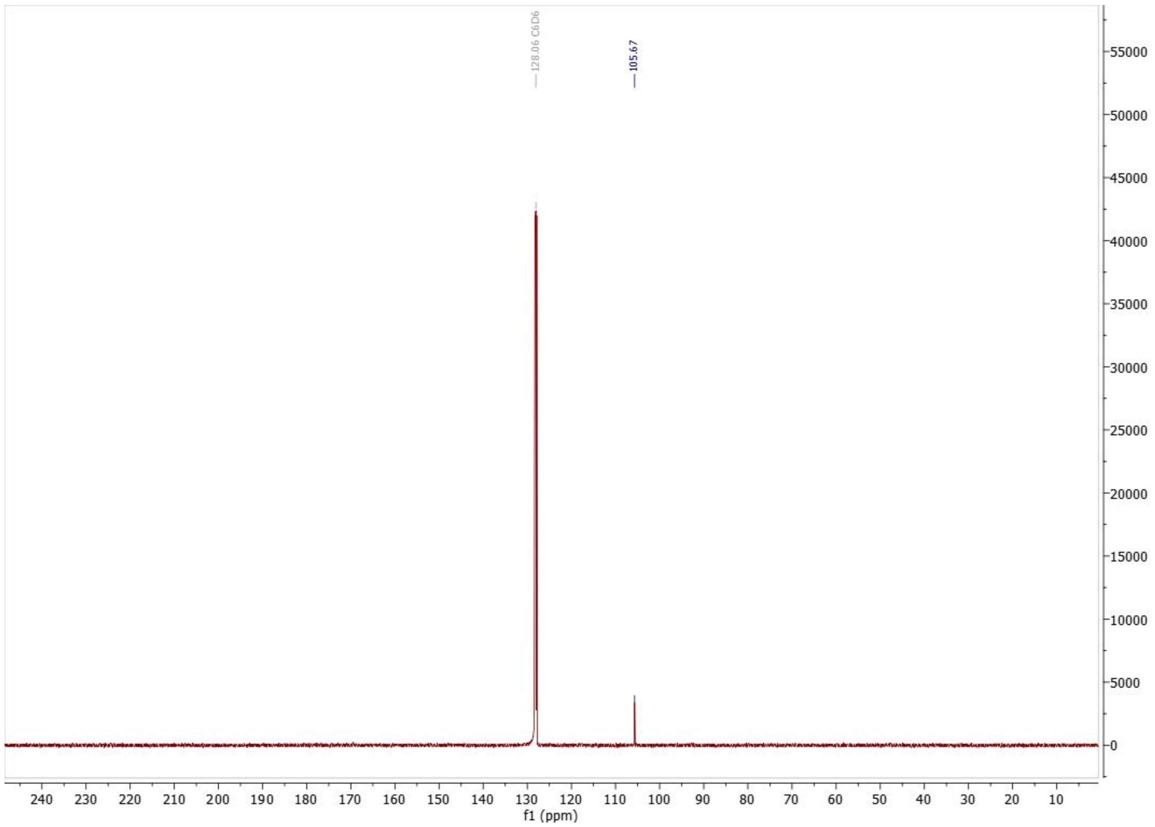

Figure S6: $^{13}C\{^{1}H\}$ NMR spectrum of **1** in d_6-benzene.

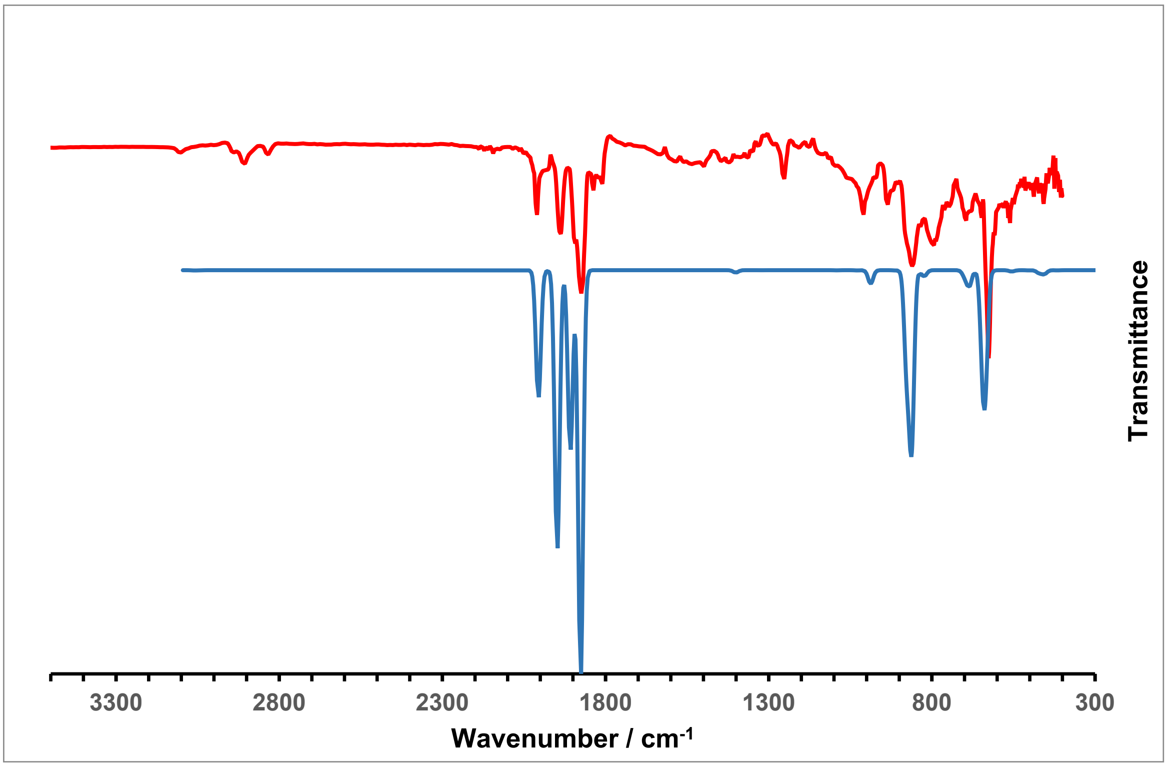

Figure S7: ATR IR spectrum of compound **2Be** (red) overlaid with the computationally simulated ATR IR spectrum for the complex (blue).

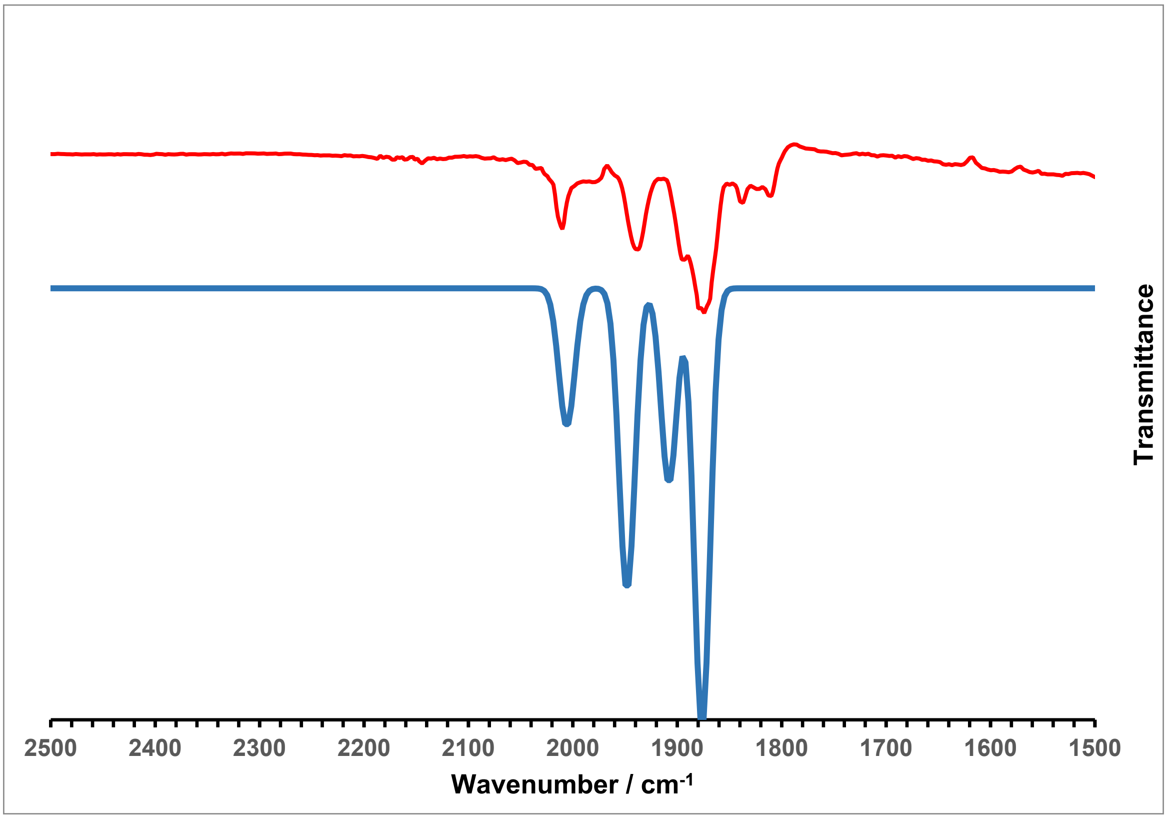

Figure S8: ATR IR spectrum (zoomed in on the carbonyl stretching region) of compound **2Be** (red) overlaid with the computationally simulated ATR IR spectrum for the complex (blue).

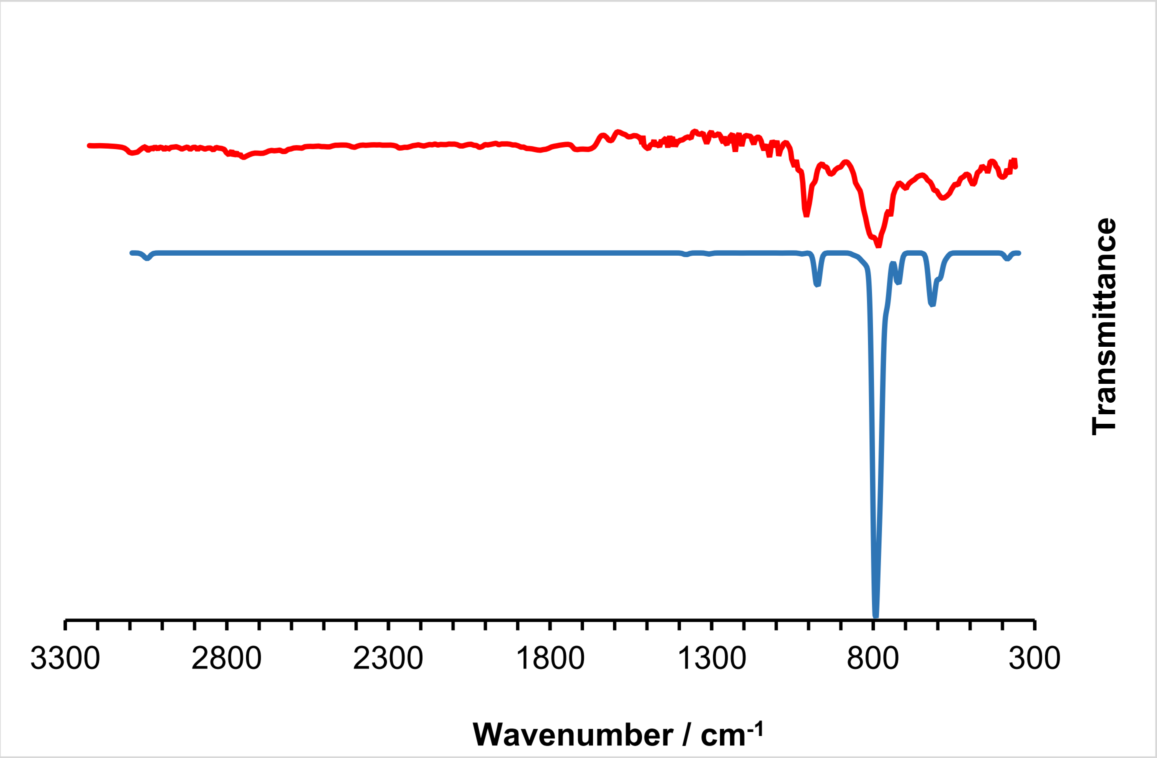

Figure S9: ATR IR spectrum of compound **1** (red) overlaid with the computationally simulated ATR IR spectrum for the complex (blue).

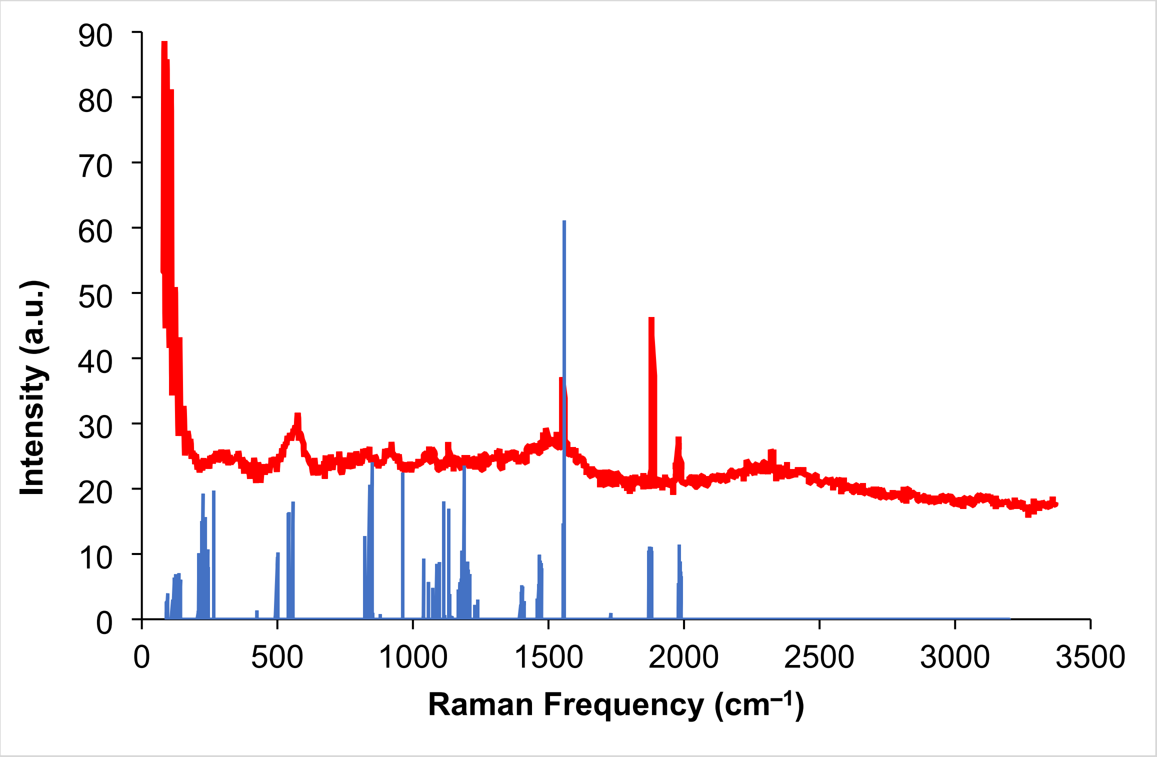

Figure S10: Raman spectrum of compound **1** (red) overlaid with the computationally simulated Raman spectrum for the complex (blue).

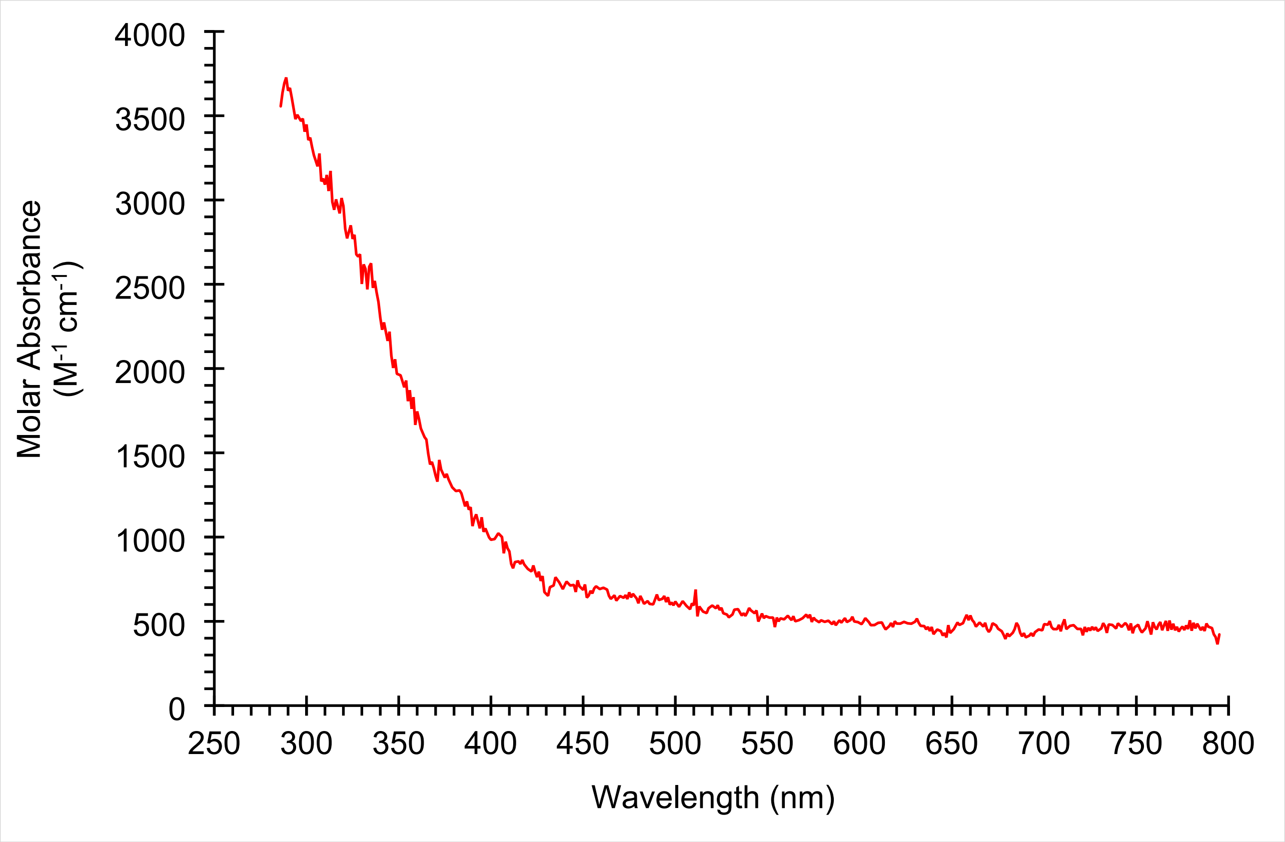

Figure S11: Optical spectrum of complex **2Be**.

Crystallographic Data:

Data for **1** and **2Be** were collected using an Oxford Diffraction/Agilent SuperNova. Crystals were selected under Paratone-N or perfluorinated oil, mounted on MiTeGen Micromount loops and quench-cooled using an Oxford Cryosystems open flow N_2 cooling device [3]. Selected details of data collection are given in Table 1. Data collected were processed using the CrysAlisPro package, including unit cell parameter refinement and inter-frame scaling (which was carried out using SCALE3 ABSPACK within CrysAlisPro) [4]. Equivalent reflections were merged and diffraction patterns processed with the CrysAlisPro suite. Structures were solved *ab initio* from the integrated intensities using SHELXT and refined on F2 using SHELXL with the graphical interface OLEX2 [5–7]. Crystallographic data is given in the supplementary deposited CIF files (CCDC 2297401-2297404) and can be obtained free of charge from the Cambridge Crystallographic Data Centre *via* http://www.ccdc.cam.ac.uk/data_request/cif.

High-quality SC XRD data for **1** was collected at both 100 and 250 K. At both temperatures the same crystallographic parameters are observed. Given the marginally higher quality of the data collected at 100 K (R-factor = 4.78, *vs* 4.81 at 250 K), data discussed in the manuscript are for this structure. Refinement of data at 100 K (without SQUEEZE) is also presented, demonstrating the disorder of the benzene over a special position and leading to poorer agreement with crystallographic data.

Table S1: Selected X-ray data collection and refinement parameters.

	**1**	**1 (w/o SQUEEZE)**	**1**	**2Be**
**Formula**	C30 H30 Be6 Ni, 3(C6 H6)	C30 H30 Be6 Ni, 3(C6 H6)	C30 H30 Be6 Ni, 3(C6 H6)	C14 H10 Be2 Fe O4
**Fw (g mol^{-1})**	737.63	737.63	737.63	316.09
**Cell setting**	hexagonal	hexagonal	hexagonal	orthorhombic
**Space group**	P 63 m c	P 63 m c	P 63 m c	-P 2ac 2n
a **(Å)**	16.0196(7)	16.0196(7)	16.25577(16)	12.7042(2)
b **(Å)**	16.0196(7)	16.0196(7)	16.25577(16)	17.3016(3)
c **(Å)**	9.1995(3)	9.1995(3)	9.30619(8)	6.44850(10)
α **(°)**	90	90	90	90
β **(°)**	90	90	90	90
γ **(°)**	120	120	120	90
V **(Å^{-3})**	2044.55(19)	2044.55(19)	2129.69(5)	1417.40(4)
Z	2	2	2	4
**ρ_{calc} (g cm^{-3})**	1.198	1.198	1.150	1.481
**Radiation, λ (Å)**	Mo K_{α}, 0.71073	Mo K_{α}, 0.71073	Cu K_{α}, 1.54184	Cu K_{α}, 1.54184
**μ (mm^{-1})**	0.506	0.506	0.865	8.611
**$R_{(int)}$**	9.37	9.37	3.14	4.82
**Parameters**	98	183	137	149
**R_1 (all data/I >2σ(I))**	0.0487/0.0479	0.0556/0.0545	0.0497/0.0481	0.0196/0.0196
**ωR_2 (all data/I >2σ(I))**	0.1395/0.1402	0.1344/0.1350	0.01387/0.1411	0.0510/0.0510
**GooF**	1.127	1.098	1.107	1.075
T **(K)**	100.01(10)	100.01(10)	249.99(10)	150.01(10)
**CCDC Deposition No.**	2297403	2297404	2297402	2297401

Computational Details:

Compounds **1** and **2Be** were optimised using ORCA (Revision 5.0.4) [8,9]. The ωB97X range-separated hybrid functional [10,11] was employed in conjunction with the Def2-QZVPP basis set [12,13] with the D4 dispersion correction [14]. The nature of the stationary points (minima) was confirmed by full frequency calculations, and are characterized by zero imaginary frequencies.

The hypothetical compound **1′** was optimised as a triplet with ORCA (Revision 5.0.4) [8,9] with the meta-generalized-gradient approximation (mGGA) functional R2-SCAN [15,16] was employed in conjunction with the Def2-TZVPPm basis set with the D4 dispersion correction [14], and employing the geometrical counterpoise correction gCP [17] (together known as the R2SCAN-3c method) [15]. In order to maintain an Octahedral geometry, constraints were applied such that all the Be-Ni-Be angles were held at 90° and Cp-Be -Ni (Cp = centroid of the Cp ring) angle was held at 180°. A single point calculation was then performed using the ωB97X range-separated hybrid functional [10,11] in conjunction with the Def2-QZVPP basis set [12,13] with the D4 dispersion correction.

In order to facilitate further analysis, single point calculations were performed using Gaussian16 (RevisionC.01) [18] using the ωB97XD [19] range-separated hybrid functional in conjunction with the Def2-QZVPP basis set [12,13] (unless otherwise specified).

Molecular Orbitals Calculated for 1:

Molecular orbitals were generated using ORCA wavefunction for the respective complex.

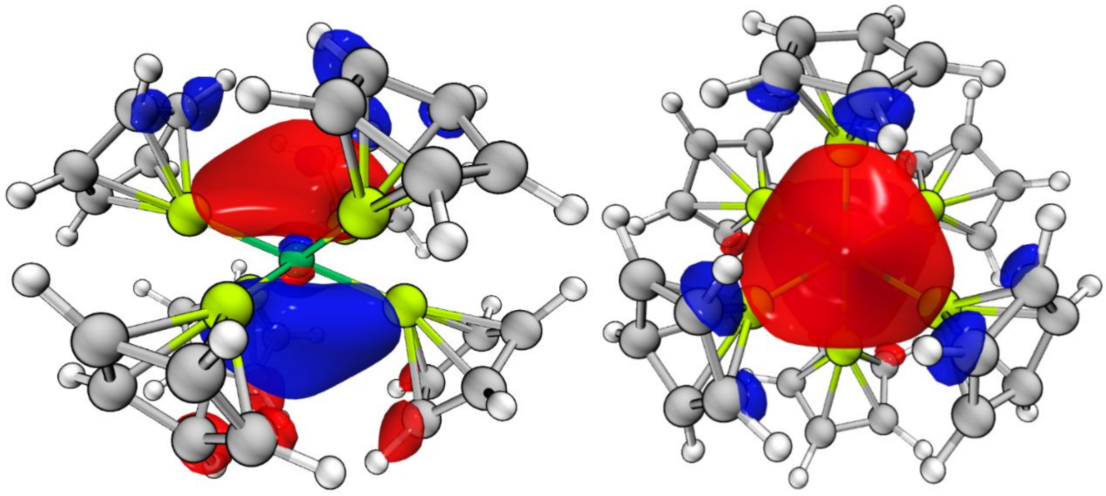

Figure S12: LUMO (0.025 a.u.) of **1** viewed perpendicular to z-axis (left) and along z-axis (right).

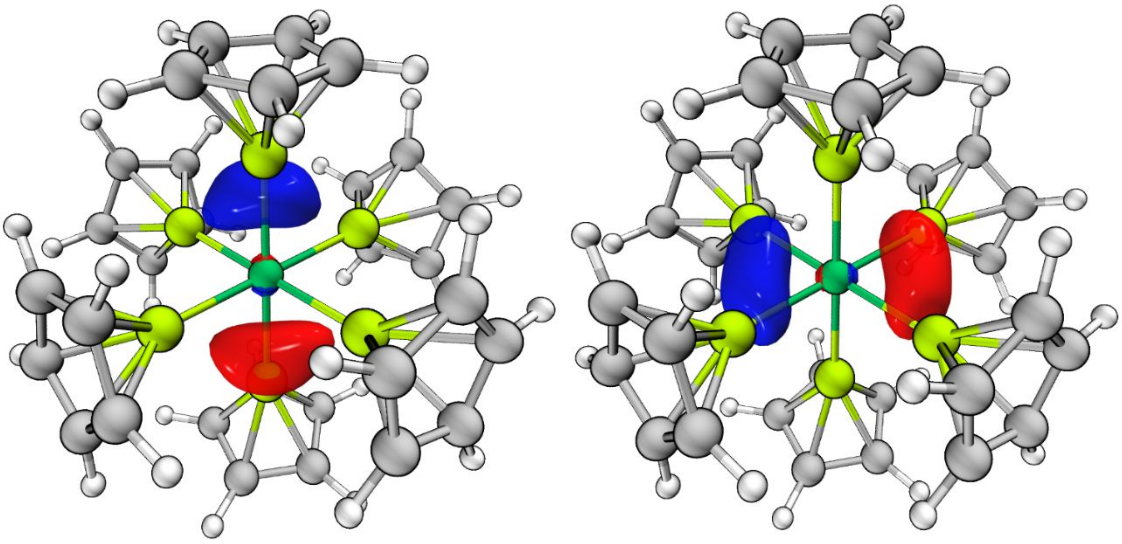

Figure S13: HOMO (left) and HOMO–1 (right) (0.075 a.u.) of complex **1**.

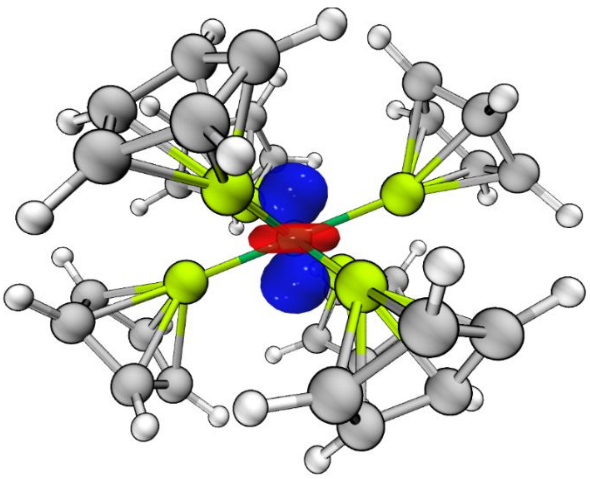

Figure S14: HOMO–2 (0.075 a.u.) of complex **1**.

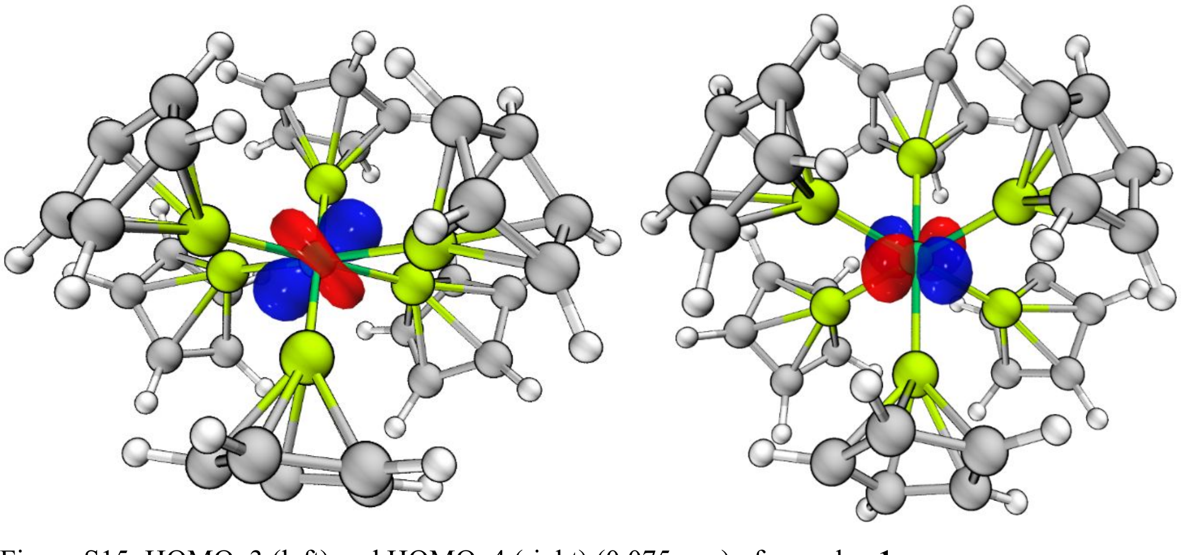

Figure S15: HOMO–3 (left) and HOMO–4 (right) (0.075 a.u.) of complex **1**.

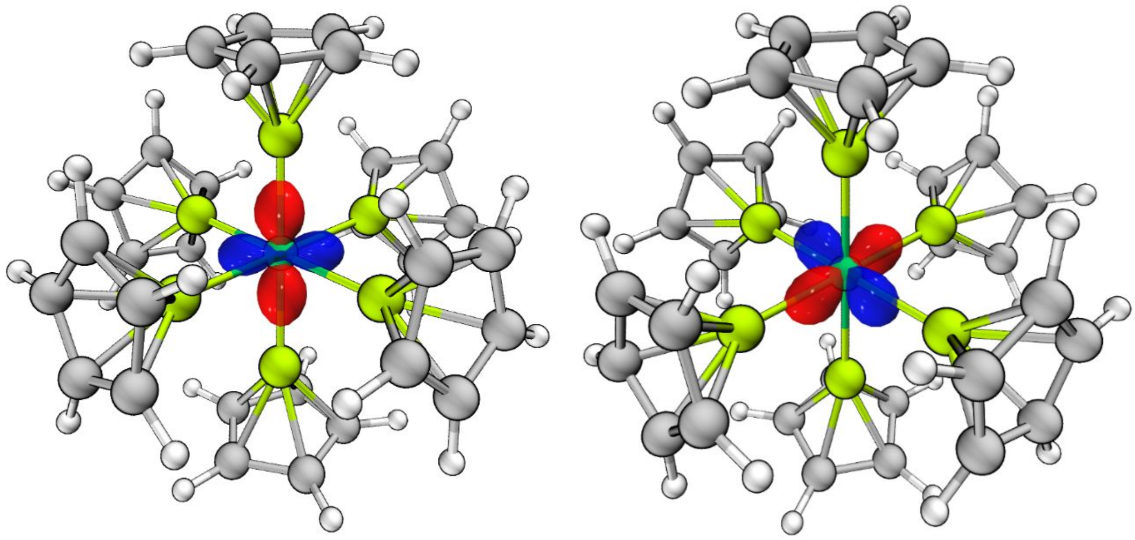

Figure S16: HOMO–5 (left) and HOMO–6 (right) (0.075 a.u.) of complex **1**.

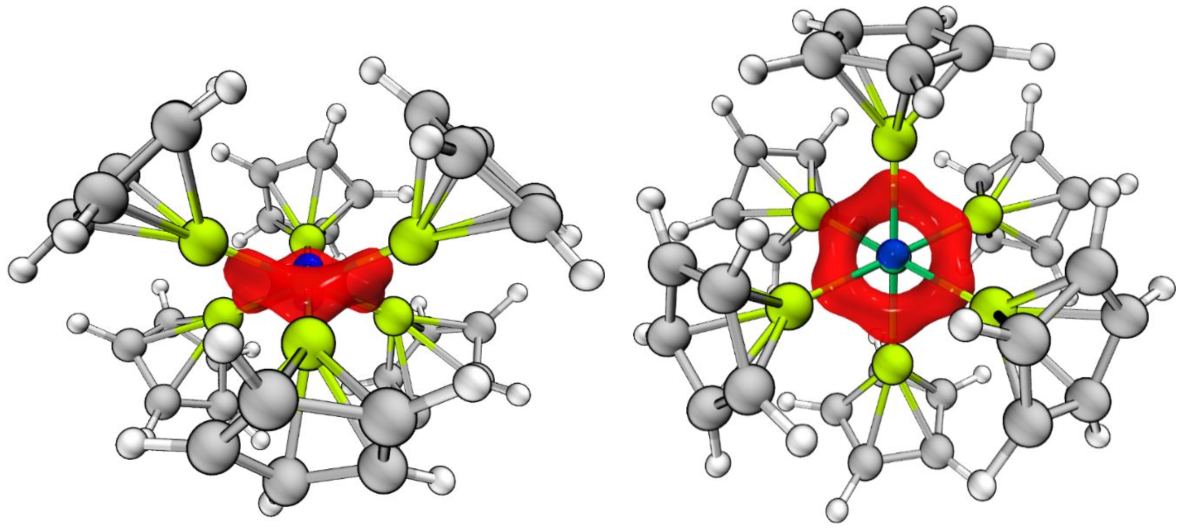

Figure S17: HOMO–19 (0.075 a.u.) of complex **1** viewed perpendicular to z-axis (left) and along z-axis (right).

Qualitative Molecular Orbital Diagram for 1 and 1′:

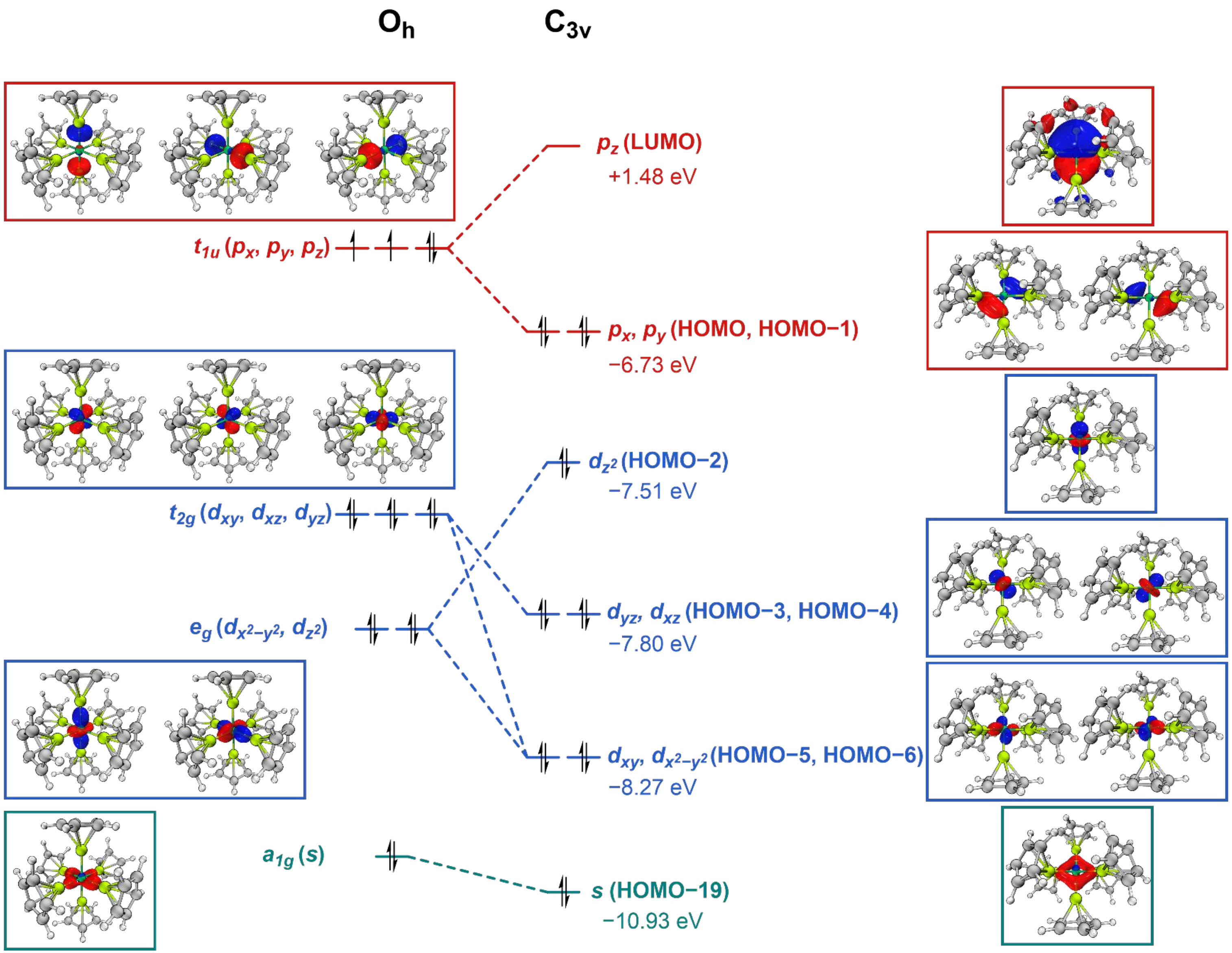

Figure S18: Qualitative molecular orbital scheme for **1′** (O_h) and **1** (C_{3v}) with corresponding molecular orbitals calculated for both complexes.

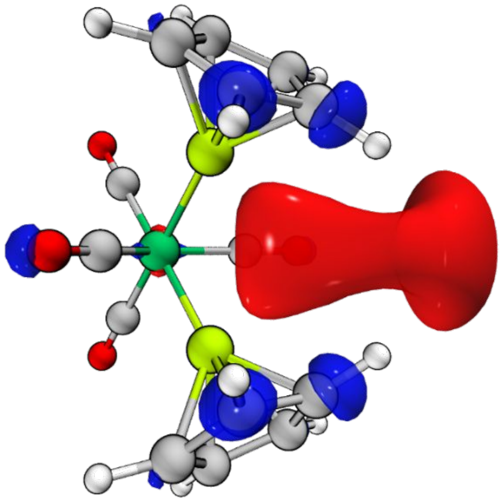

Figure S19: LUMO (0.025 a.u.) of complex **2Be**.

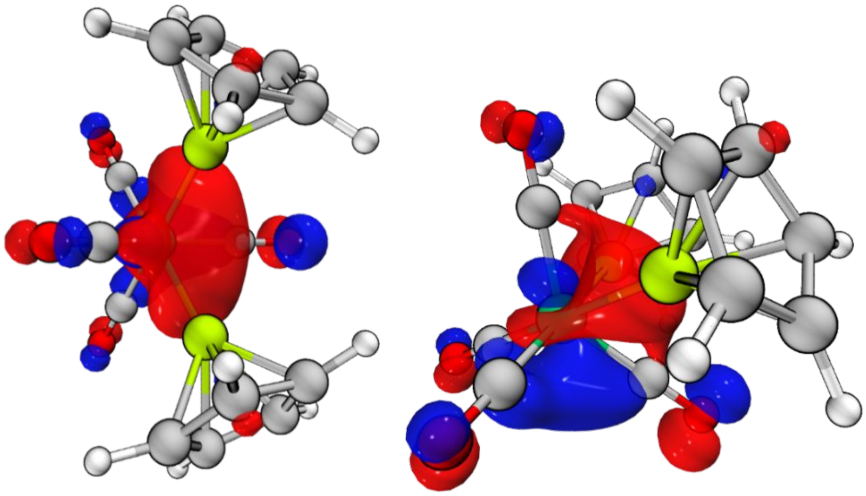

Figure S20: HOMO (0.05 a.u.) of complex **2Be** viewed with the Fe–Be bonds in the plane (left) and from the side (right).

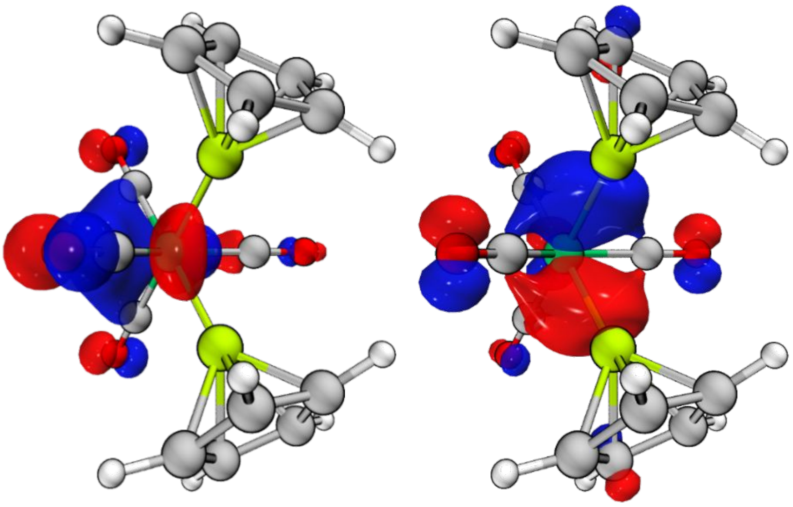

Figure S21: HOMO–1 (left) and HOMO–2 (right) (0.05 a.u.) of complex **2Be**.

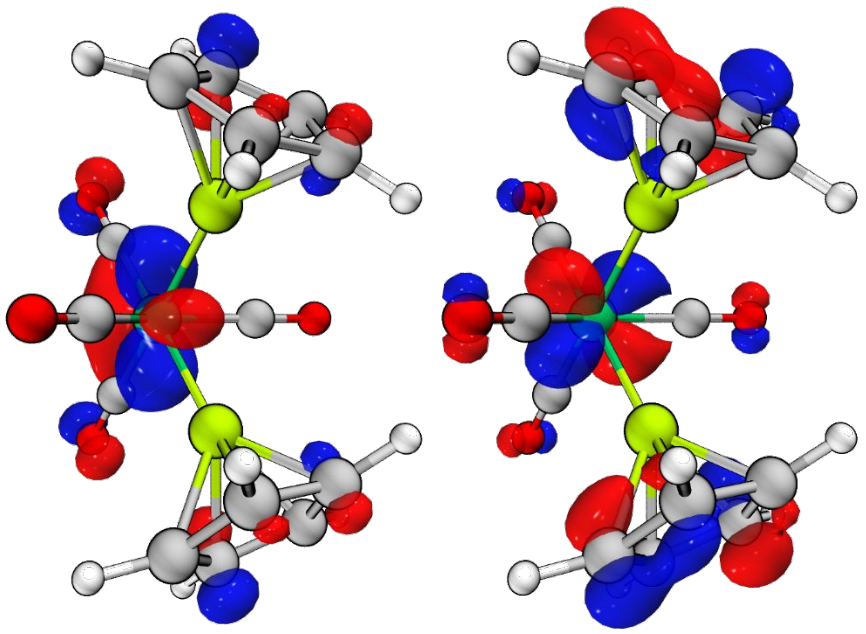

Figure S22: HOMO–3 (left) and HOMO–4 (right) (0.05 a.u.) of complex **2Be**.

Quantum Theory of Atoms in Molecules (QTAIM):

QTAIM was preformed using the AIMALL programme [20]. Calculations were performed using the ORCA wavefunctions for the respective complexes.

Table S2: Summary of QTAIM data generated for **1**. All values in a.u., unless specified. ELF and LOL are unit-less.

**Ni–Be BCP**	**101**	**124**	**128**	**138**	**142**	**164**	**Average**
ρ_{bcp} / e^{-} Bohr^{-3}	0.06078	0.06097	0.06093	0.06091	0.06092	0.06073	0.06087
**KE (L)**	0.02944	0.02982	0.02967	0.02958	0.02971	0.02944	0.02961
**KE (H)**	0.03316	0.03330	0.03327	0.03327	0.03327	0.03313	0.03323
**V**	-0.06260	-0.06311	-0.06294	-0.06284	-0.06297	-0.06257	-0.06284
**E**	-0.03316	-0.03330	-0.03327	-0.03327	-0.03327	-0.03313	-0.03323
$\nabla^2\rho_{bcp}$ / e^{-} Bohr^{-5}	-0.01489	-0.01392	-0.01443	-0.01475	-0.01424	-0.01475	-0.01450
**ELF**	0.45622	0.45247	0.45446	0.45572	0.45366	0.45565	0.45470
**LOL**	0.47815	0.47626	0.47727	0.47790	0.47687	0.47787	0.47739

Table S3: Summary of QTAIM data generated for **1′**. All values in a.u., unless specified. ELF and LOL are unit-less.

**Ni–Be BCP**	**101**	**124**	**128**	**138**	**142**	**164**	**Average**
ρ_{bcp} / e^{-} Bohr^{-3}	0.05208	0.05215	0.06735	0.05610	0.05600	0.06712	0.05847
**KE (L)**	0.03286	0.03299	0.03917	0.04136	0.04114	0.03865	0.03770
**KE (H)**	0.02763	0.02766	0.03790	0.02952	0.02948	0.03779	0.03166
**V**	-0.06049	-0.06065	-0.07707	-0.07088	-0.07062	-0.07643	-0.06936
**E**	-0.02763	-0.02766	-0.03790	-0.02952	-0.02948	-0.03779	-0.03166
$\nabla^2\rho_{bcp}$ / e^{-} Bohr^{-5}	0.02092	0.02134	0.00509	0.04738	0.04666	0.00344	0.02414
**ELF**	0.32562	0.32462	0.40047	0.27826	0.27936	0.40421	0.33542
**LOL**	0.40992	0.40937	0.44975	0.38307	0.38371	0.45167	0.41458

Table S4: Summary of QTAIM data generated for **2Be**. All values in a.u., unless specified. ELF and LOL are unit-less.

	**Axial CO**		**Equatorial CO**		**Be**	
**Fe-L BCP**	**49**	**50**	**48**	**52**	**46**	**54**
ρ_{bcp} / e^{-} Bohr^{-3}	0.16516	0.16539	0.15294	0.15294	0.05628	0.05628
**KE (L)**	0.22287	0.22206	0.21159	0.21159	0.03279	0.03278
**KE (H)**	0.09010	0.09051	0.07464	0.07464	0.02866	0.02866
**V**	-0.31298	-0.31257	-0.28623	-0.28623	-0.06145	-0.06145
**E**	-0.09010	-0.09051	-0.07464	-0.07464	-0.02866	-0.02866
$\nabla^2\rho_{bcp}$ / e^{-} Bohr^{-5}	0.53108	0.52621	0.54783	0.54783	0.01651	0.01649
**ELF**	0.29086	0.29336	0.26048	0.26048	0.34360	0.34365
**LOL**	0.39042	0.39186	0.37246	0.37246	0.41986	0.41989

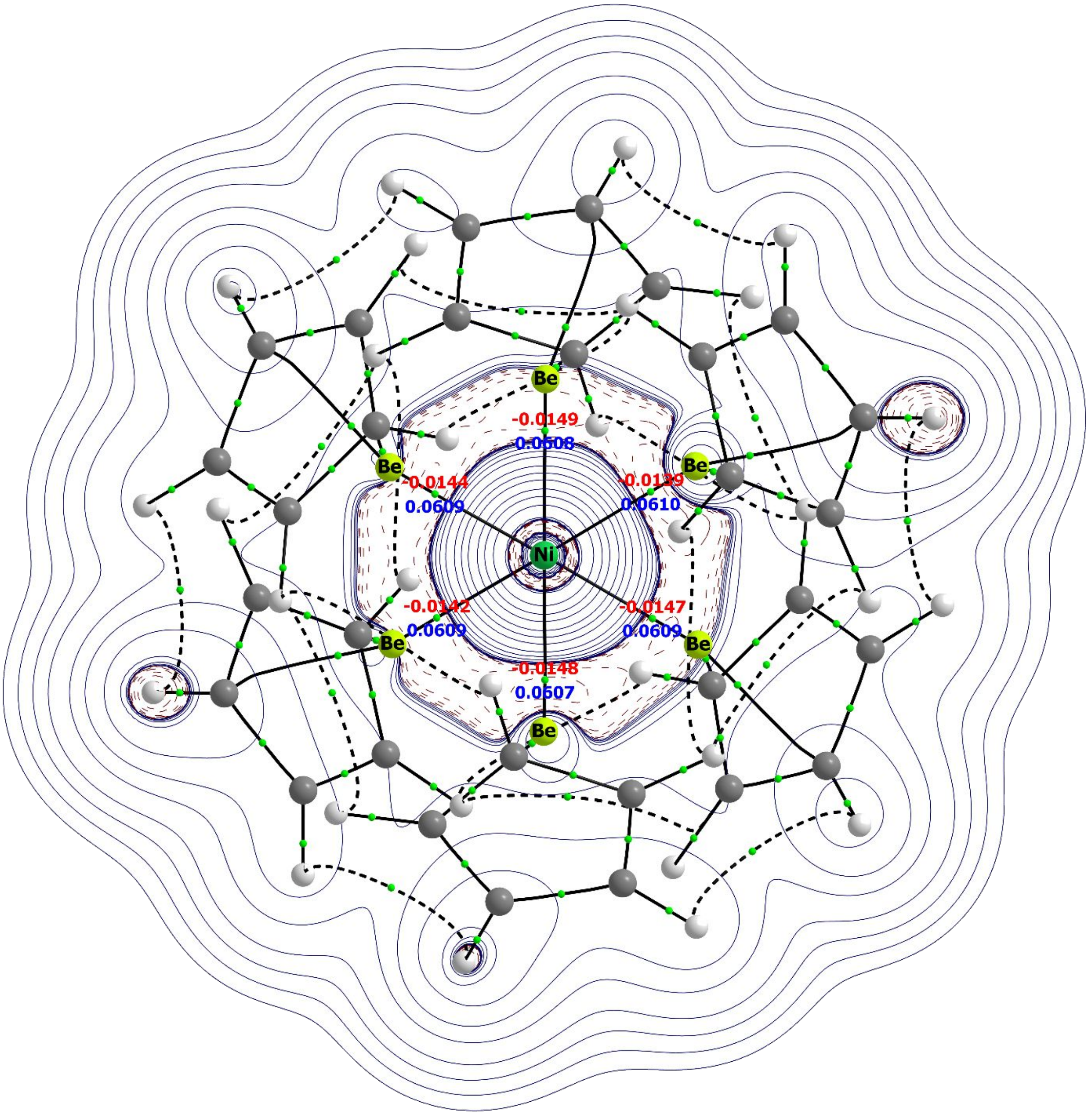

Figure S23: Plot of QTAIM data generated for **1**. Bond paths (BPs) in black and bond critical points (BCPs) in green. Contour map of is of Laplacian ($\nabla^2\rho_{bcp}$). Values in red are $\nabla^2\rho_{bcp}$ (e^- Bohr^{-5}) and values in blue are ρ_{bcp} (electron density; e^- Bohr^{-3}) at the respective BCP. The negative $\nabla^2\rho_{bcp}$ values are indicative of covalent Ni–Be bonding, which contrasts with **1′** (see below). Ring critical points and prismatic critical points are omitted for clarity.

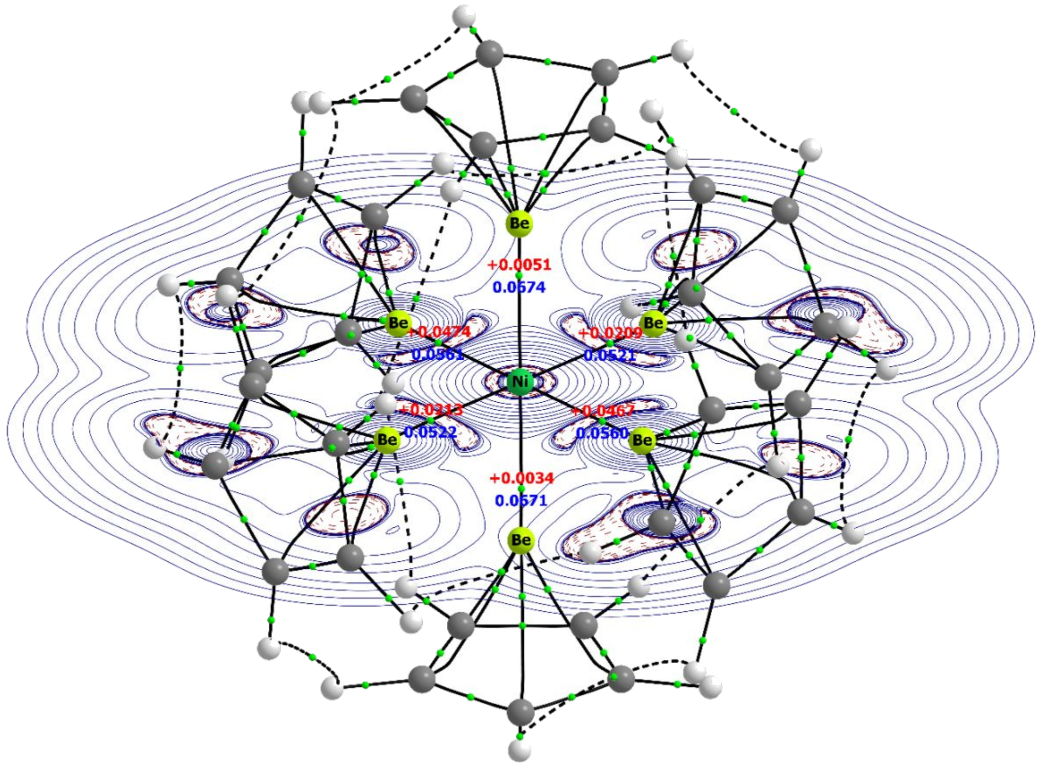

Figure S24: Plot of QTAIM data generated for **1′**. Bond paths (BPs) in black and bond critical points (BCPs) in green. Contour map of is of Laplacian ($\nabla^2\rho_{bcp}$). Values in red are $\nabla^2\rho_{bcp}$ (e^- Bohr^{-5}) and values in blue are ρ_{bcp} (electron density; e^- Bohr^{-3}) at the respective BCP. The positive values of $\nabla^2\rho_{bcp}$ are indicative that the bonding is ionic in nature. Ring critical points and prismatic critical points are omitted for clarity.

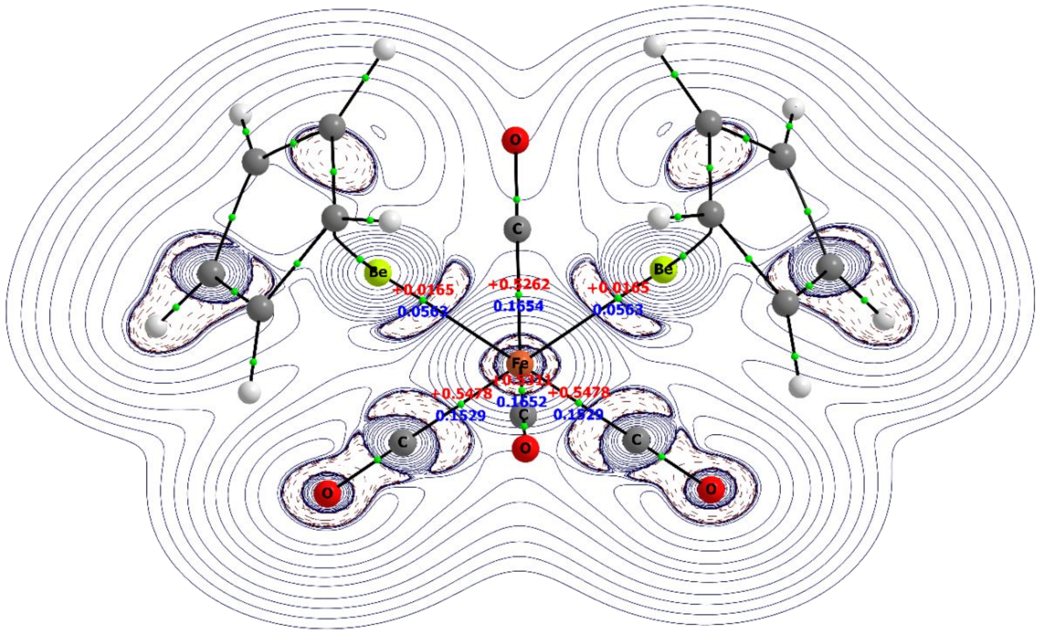

Figure S25: Plot of QTAIM data generated for **2Be**. Bond paths (BPs) in black and bond critical points (BCPs) in green. Contour map of is of Laplacian ($\nabla^2\rho_{bcp}$). Values in red are $\nabla^2\rho_{bcp}$ (e^- Bohr^{-5}) and values in blue are ρ_{bcp} (electron density; e^- Bohr^{-3}) at the respective BCP. Ring critical points and prismatic critical points are omitted for clarity.

Localised Orbital Locator (LOL) and Electron Localisation Function (ELF) Calculations:

Calculations were performed using the ORCA wavefunctions for the respective complexes, generated using Multiwfn 3.8 [21–23]. The toroidal feature in the ELF isosurface for **1** comprises six adjacent $NiBe_2$ tri-synaptic basins. The co-variance values support delocalisation across these six basins [24]. The sum of the electrons across these basins is approximately 10 electrons, as would be expected from the five Ni–Be bonding orbitals (2 x 3*d*, 2 x 4*p*, 1 x 4*s*). LOL calculations yield a similar picture to that of the ELF calculations.

Table S5: Summary of ELF data for **1**.

**Attractors**	**20(t)**	**21(t)**	**22(t)**	**37(t)**	**41(t)**	**45(t)**
**population**	1.3946	1.4142	1.3946	1.4109	1.4151	1.3977
**volume**	8.693	8.881	8.731	8.865	8.884	8.736
**LI[a]**	0.43995	0.45094	0.43917	0.44909	0.45072	0.44132
**DI[b]**	0.27963049	0.27963049	0.27972373	0.2794927	0.2799513	0.27972162
**(X,Y)**	20,21	21,20	22,21	37,20	41,22	45,37
**DI[b]**	0.2794927	0.27972373	0.2799513	0.27972162	0.28016953	0.28016953
**(X,Y)**	20,37	21,22	22,41	37,45	41,45	45,41

a) LI = localization index; b) DI = delocalization index.

Table S6: Summary of LOL data for **1**.

**Attractors**	**35**	**37**	**38**	**39**	**40**	**41**
**integration**	1.568249953	1.607928229	1.560850544	1.571359302	1.613237857	1.606682995
**volume**	66.690216	68.773752	66.451104	67.000824	69.1362	68.545872
**LI[a]**	0.49347	0.51676	0.48912	0.4942	0.51948	0.51624
**DI[b]**	0.31480287	0.31480287	0.31481624	0.31570007	0.31591996	0.31520429
**(X,Y)**	35,37	37,39	38,40	39,37	40,35	41,38
**DI[b]**	0.31591996	0.31570007	0.31520429	0.3155519	0.31481624	0.3155519
**(X,Y)**	35,40	37,39	38,41	39,41	40,38	41,39

a) LI = localization index; b) DI = delocalization index.

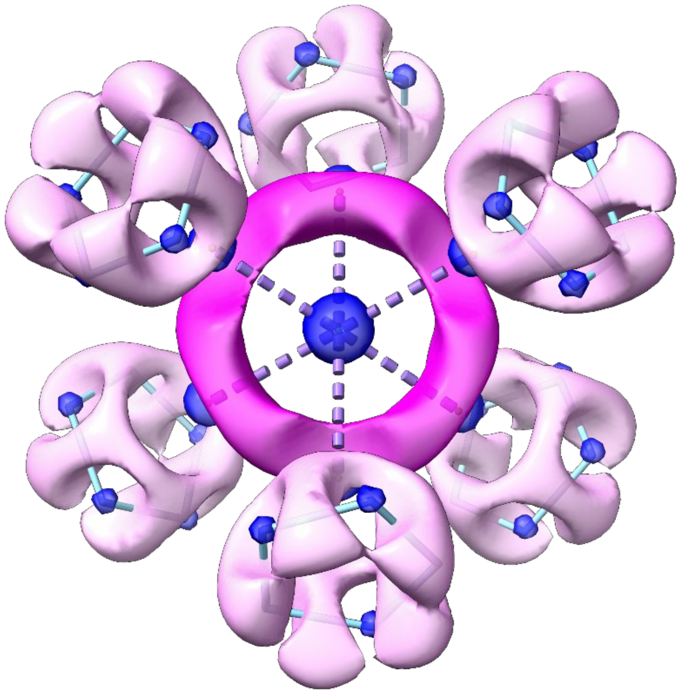

Figure S26: ELF isosurface for **1** (with H atoms omitted for clarity) (0.7 a.u.). Pink represents the isosurfaces associated with the Cp ligands. Magenta represents the isosurface associated with the Be–Ni tri-synaptic basins, which contain an average 1.37 electrons (Ni:Be:Be 74:13:13).

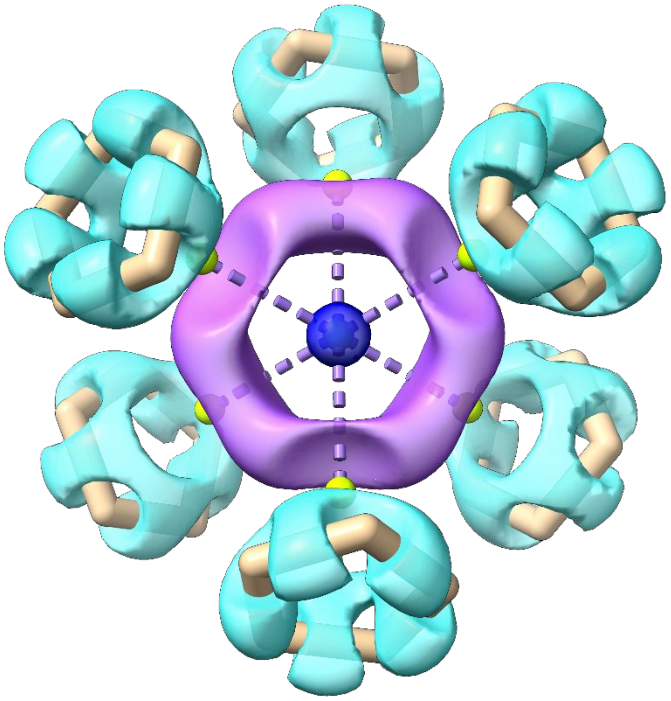

Figure S27: LOL isosurface for **1** (with H atoms omitted for clarity), viewed along z-axis (0.5 a.u.). Cyan represents the isosurfaces associated with Cp ligand basins. Be–Ni tri-synaptic basins contain an average 1.52= electrons (Ni:Be:Be 74:13:13).

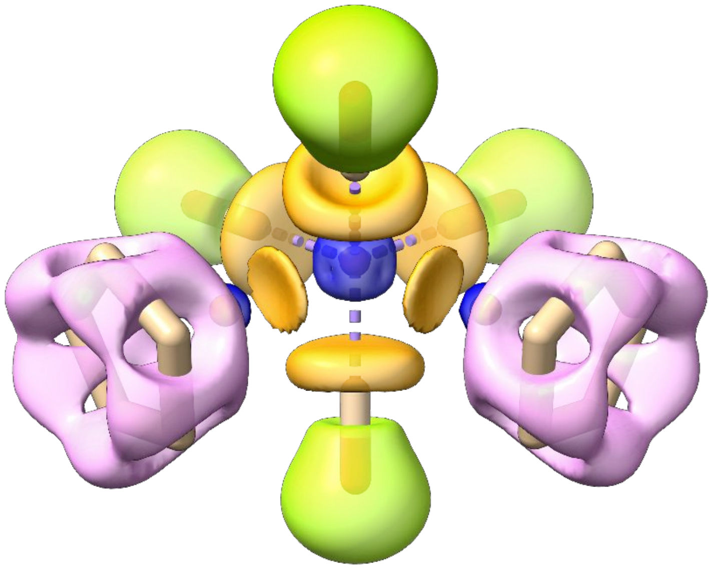

Figure S28: ELF isosurface for **2Be** (with H atoms omitted for clarity) (0.7 a.u.). Pink represents the isosurfaces associated with the Cp ligands. Orange represents the Fe–ligand disynaptic basin isosurfaces. Dark blue are isosurfaces of the core basins. Fe–Be disynaptic basins each contain 1.07 electrons (Fe:Be 79:21).

Natural Bond Orbital (NBO) Analysis:

NBO calculations were performed on the ORCA wavefunction for **1** using NBO 7.0 [25] to compliment QTAIM and ELF/LOL calculations. Although Be–Ni bonds are not found by this programme, Wiberg bond indices of 0.27 for the six Be–Ni bonds are calculated. Natural charges for Ni (–1.14) and the six Be centres (+1.04) are consistent with charge transfer from Be to Ni.

Selected Adaptive natural density partitioning (AdNDP) orbitals:

Adaptive natural density partitioning (AdNDP) analysis of the wavefunction produced by Gaussian 16 was performed on **1** using the Multiwfn code [26,27]. This technique, which is effective at describing electron delocalisation, has been widely employed for the examination of aromaticity in organic and inorganic systems. Aside from the expected bonding interactions associated with the Cp σ- and π-bonding frameworks and other Be–Ni σ-bonding combinations (fig. S27-S30), AdNDP returns a delocalised $NiBe_6$ σ-symmetry bonding orbital (fig. S31).

Table S7: Summary of AdNDP orbital composition for **1**.

	**orbital**	**1**	**2**	**3**	**4**	**5**	**6**	**7**	**8**	**9**
**Ni**	d_{xy}	3.4%	5.6%		44.9%	43.3%				
	d_{xz}	62.1%	27.8%		1.8%	7.8%				
	d_{yz}	27.7%	62.5%		7.4%	1.9%				
	$d_{x^2-y^2}$	5.8%	3.1%		43.5%	44.7%				
	d_{z^2}			99.3%						
	4_p							56.0%	56.0%	71.2%
	4s						49.9%			
	**Total Ni**	99.1%	99.1%	99.3%	97.8%	97.7%	49.9%	56.0%	56.0%	71.2%
**Be**	**1: 2*s***						8.3%	11.0%	3.7%	4.8%
	**2: 2*s***				0.5%		8.3%	10.6%	4.1%	4.8%
	**3: 2*s***						8.3%	11.0%	3.6%	4.8%
	**4: 2*s***				0.5%		8.3%	10.6%	4.0%	4.8%
	**5: 2*s***					0.5%	8.5%		14.3%	4.8%
	**6: 2*s***					0.6%	8.4%		14.3%	4.8%
	**Total Be**				1.0%	1.1%	50.1%	43.3%	44.0%	28.8%
	**O.N.**	2.00	2.00	2.00	2.00	2.00	1.99	1.97	1.97	0.32
	**energy / eV**	-7.90	-7.88	-7.52	-7.94	-7.94	-18.36	-9.70	-9.70	-14.32

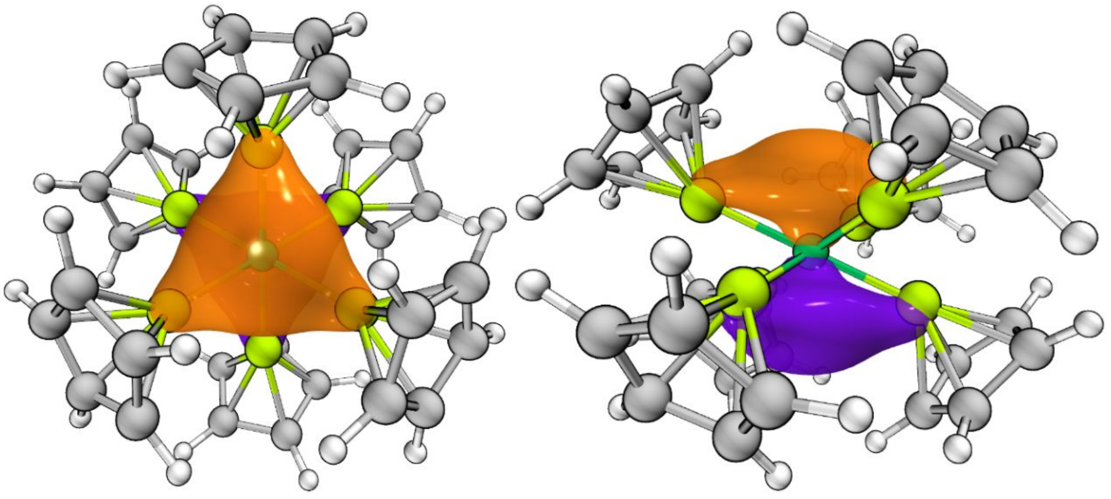

Figure S29: AdNDP orbital 9 (essentially the LUMO) for **1** viewed along the z-axis (left) and perpendicular to the z-axis (right) (0.5 a.u.).

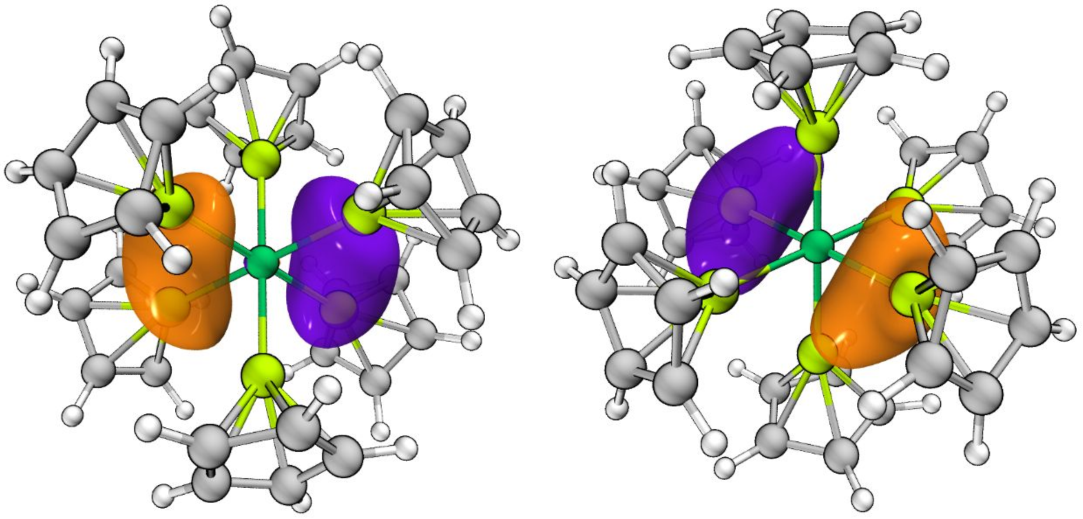

Figure S30: AdNDP orbitals for **1**. Orbital 7 (essentially the HOMO), left; orbital 8 (essentially the HOMO-1), right (0.5 a.u.).

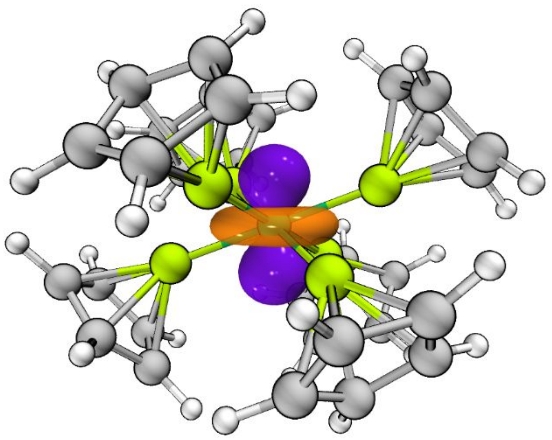

Figure S31: AdNDP orbital 3 (essentially HOMO-2) for **1** (0.5 a.u.).

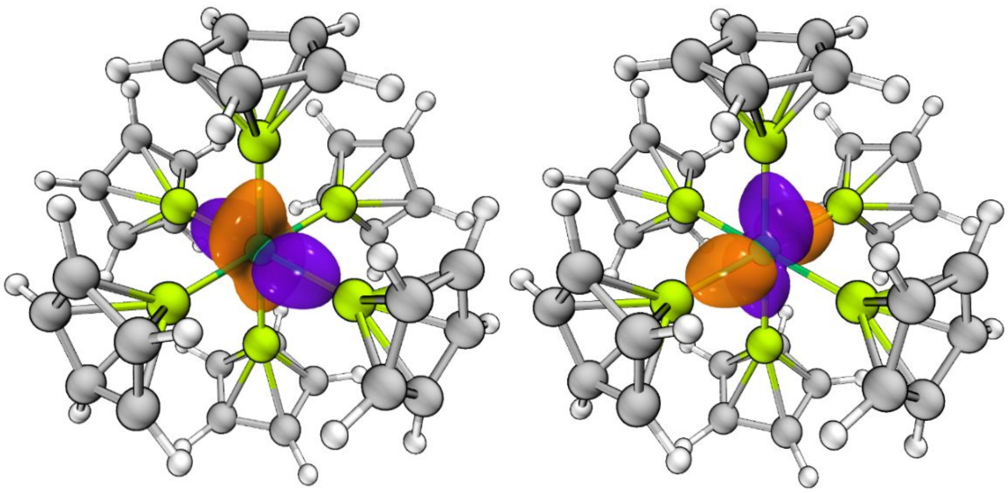

Figure S32: AdNDP orbitals for **1**. Orbital 1 (essentially HOMO-3), left; orbital 2 (essentially HOMO-4), right (0.5 a.u.).

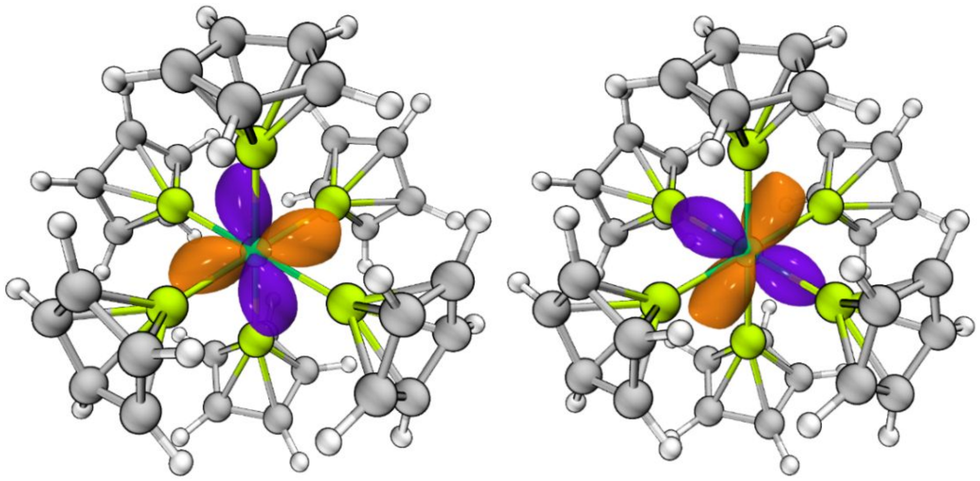

Figure S33: AdNDP orbitals for **1**. Orbital 4 (essentially HOMO-5), left; orbital 5 (essentially HOMO-6), right (0.5 a.u.).

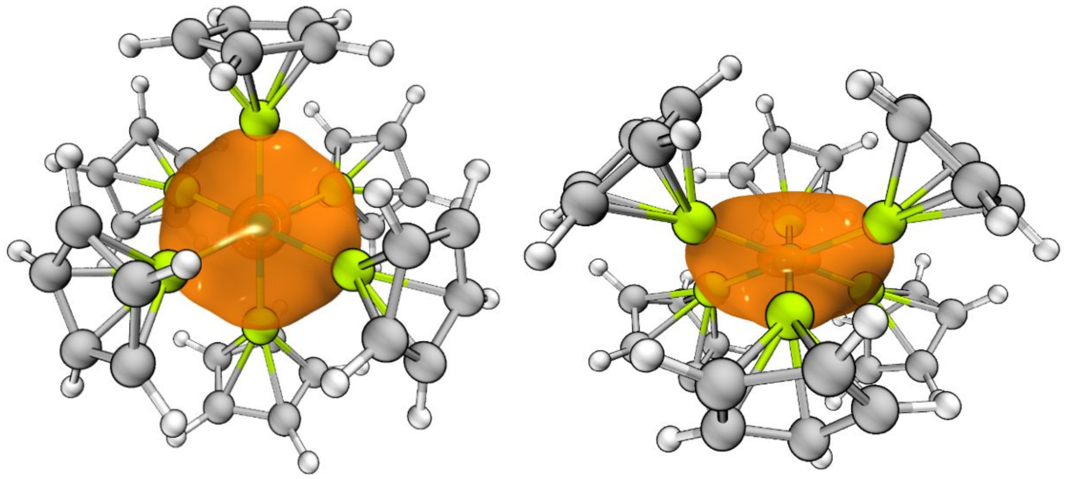

Figure S34: AdNDP orbital 6 (essentially HOMO-19) for **1** viewed along the z-axis (left) and perpendicular to the z-axis (right) (O.N. = 1.9) (0.5 a.u.). This orbital comprises contributions from the Ni 4*s*-orbital (49.9%) and each of the 2*s*-orbitals of the six beryllium atoms (8.26 – 8.47% from each) (Table S7).

Electron Density of Delocalised Bonds (EDDB) Analysis:

EDDB analysis of the wavefunction produced by Gaussian 16 was performed on **1** using the RunEDDB code [28]. A global search finds 38.9928 delocalised electrons of 262.0000 NPA electrons (Table S8).

Table S8: Summary of EDDB data for electron delocalisation across the $NiBe_6$ unit.

**Atom**	Be	Be	Be	Be	Be	Be	Ni
**Number of delocalised electrons**	0.2709	0.2700	0.2704	0.2706	0.2705	0.2701	1.6321

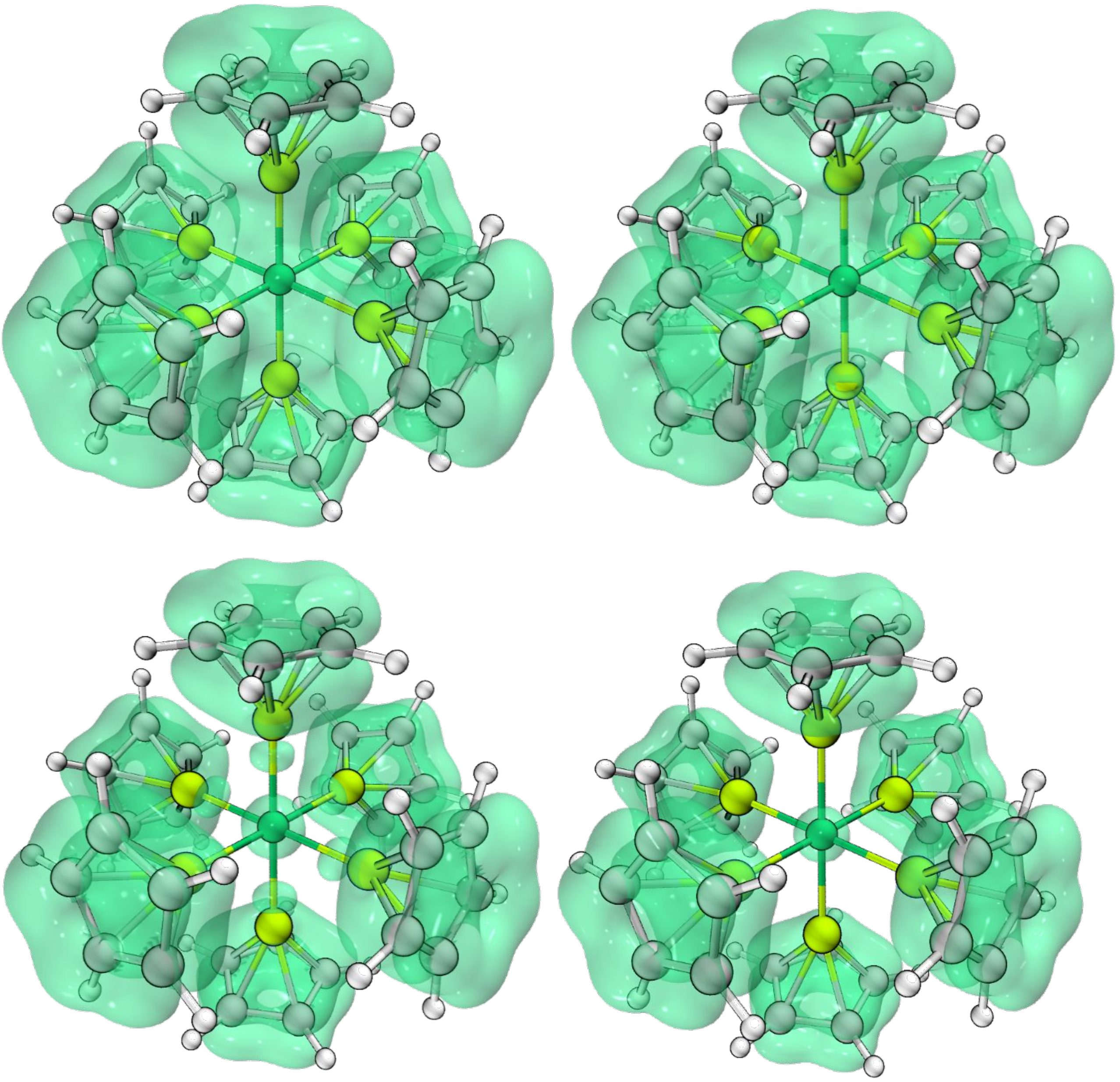

Figure S35: EDDB isosurface for **1** (top left, 0.01 a.u.; top right, 0.00125 a.u.; bottom left 0.015 a.u.; bottom right, 0.02 a.u.)

Nucleus-Independent Chemical Shift (NICS) Analysis:

NICS analysis was performed in Gaussian 16 (Gaussian wavefunction) using the GIAO method [29]. Isochemical shielding surface (ICSS) analysis was performed using the M06-2x hybrid functional with Def2-TZVP [12,13].

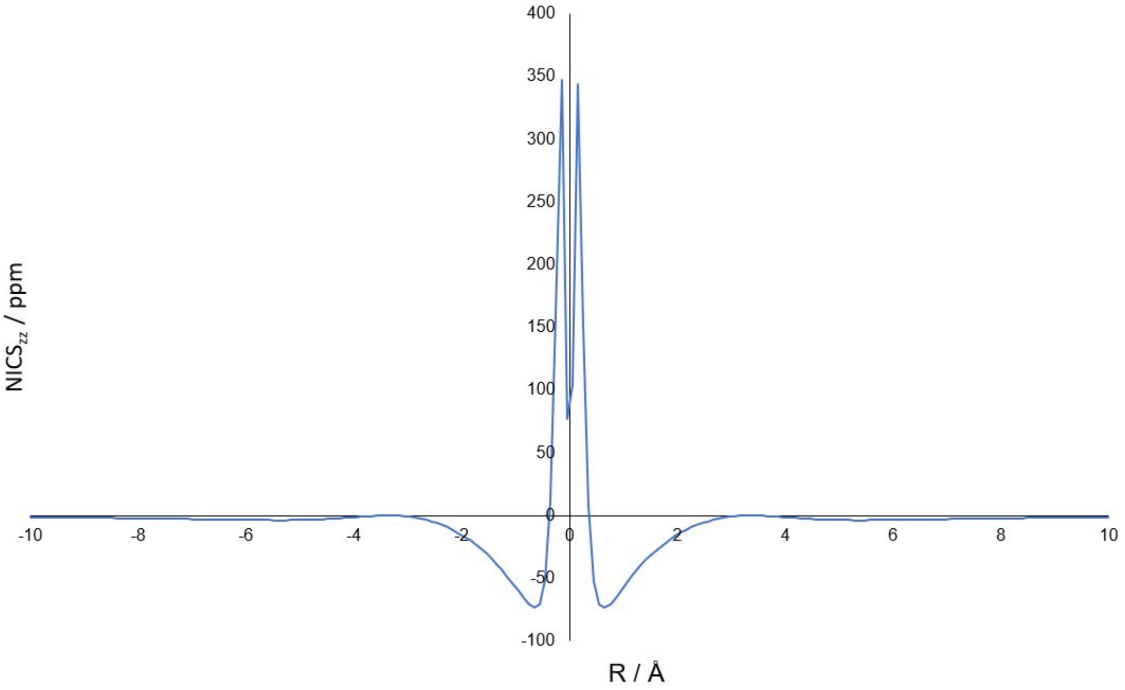

Figure S36: $NICS_{zz}$ plot for complex **1**. R is the distance along the C_3 axis from the Ni centre.

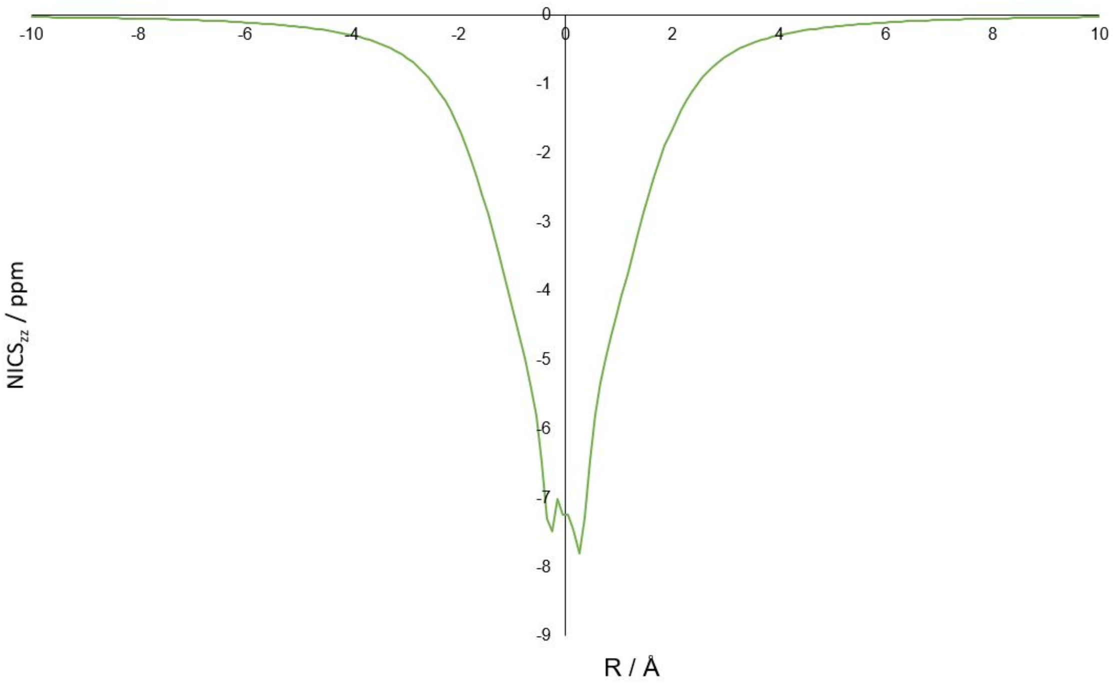

Figure S37: $NICS_{zz}$ plot for the HOMO−19 of complex **1**. R is the distance along the C_3 axis from the Ni centre.

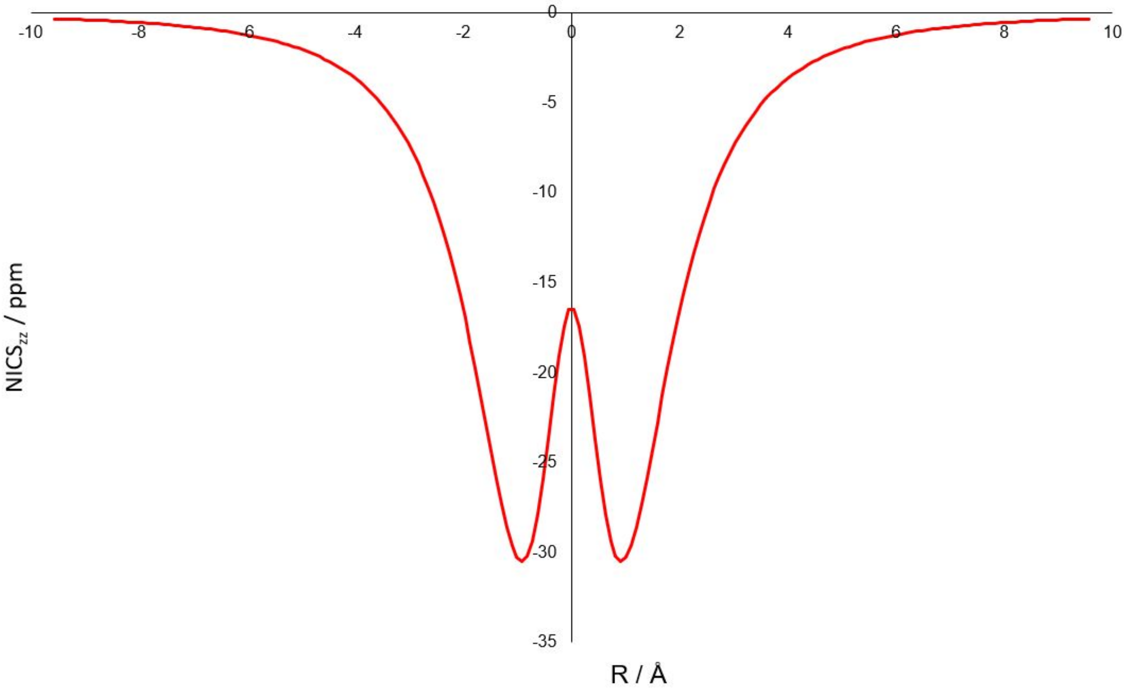

Figure S38: $NICS_{zz}$ plot for benzene. R is the perpendicular distance from the C_6 ring centroid.

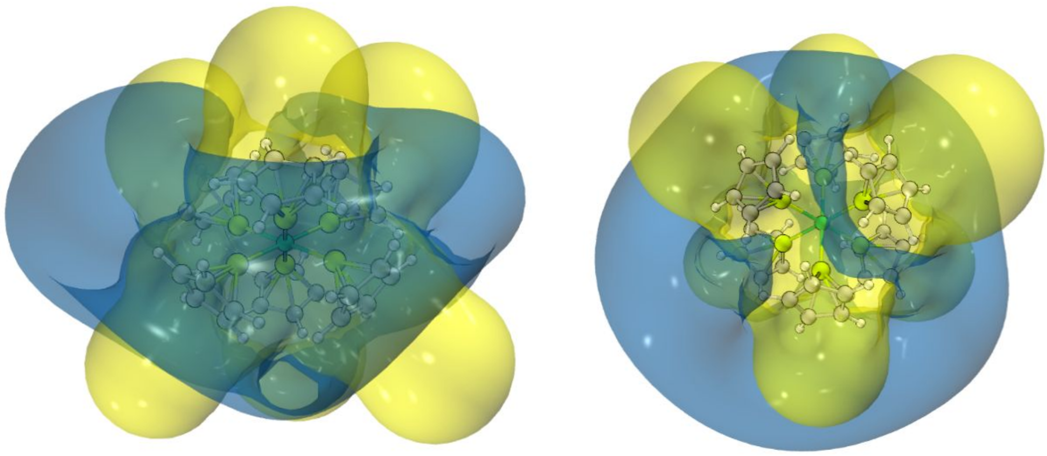

Figure S39: Isotropic isochemical shielding surface plots for **1** viewed perpendicular to the z-axis (left) and along the z-axis (right) (0.75 ppm).

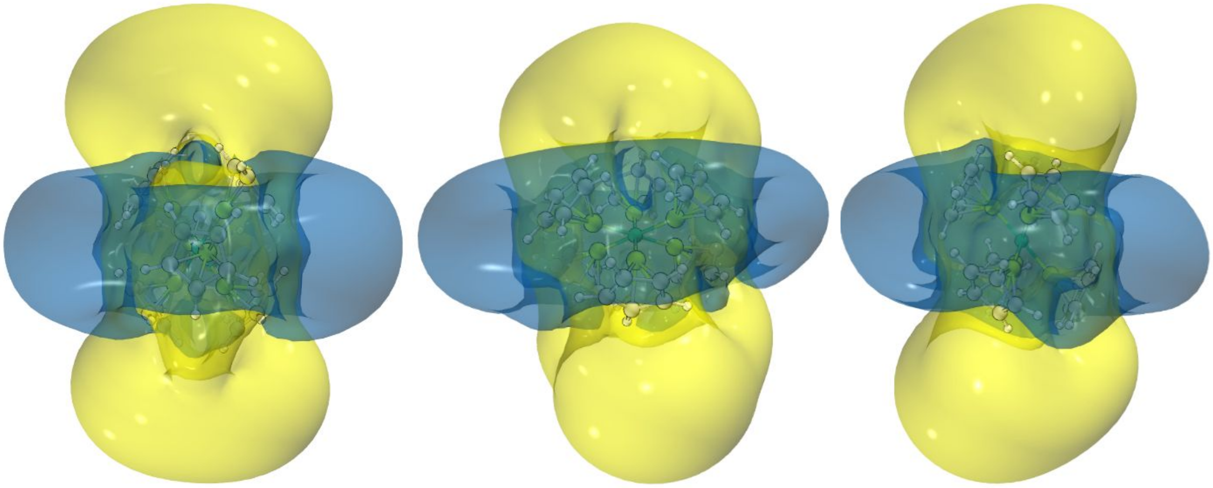

Figure S40: Isochemical shielding surface plots for **1**, $ICSS_{xx}$ (left), $ICSS_{yy}$ (centre), $ICSS_{zz}$ (right) (1 ppm).

QTAIM Magnetic Analysis:

Magnetic analysis was performed using AIMALL using the wavefunction generated by Gaussian 16 (GIAO method) [20].

Table S9: Magnetizability data calculated for **1**. Units of χ are ppm.

**Atom**	χ^{iso}**Intra**	χ^{iso}**Bond**	χ^{iso}	χ^{zz}**Intra**	χ^{zz}**Bond**	χ^{zz}
Ni	-24.65	-5.55	-30.20	-45.98	-9.95	-55.93
Be	-0.36	-0.20	-0.56	-0.14	-0.72	-0.87
Be	-0.36	-1.41	-1.77	-0.14	-2.20	-2.34
Be	-0.36	-2.16	-2.52	-0.14	-2.50	-2.65
Be	-0.36	-0.90	-1.26	-0.14	-0.13	-0.27
Be	-0.36	-1.62	-1.98	-0.14	-1.21	-1.36
Be	-0.36	-0.61	-0.97	-0.14	-0.14	-0.28
**Av. Be**	-0.36	-1.15	-1.51	-0.14	-1.15	-1.29

It should be noted that χ_{zz}^{Atom} for Be would be expected to be smaller than those of benzene as the Be centres are not within the space defined by the delocalised ELF isosurface.

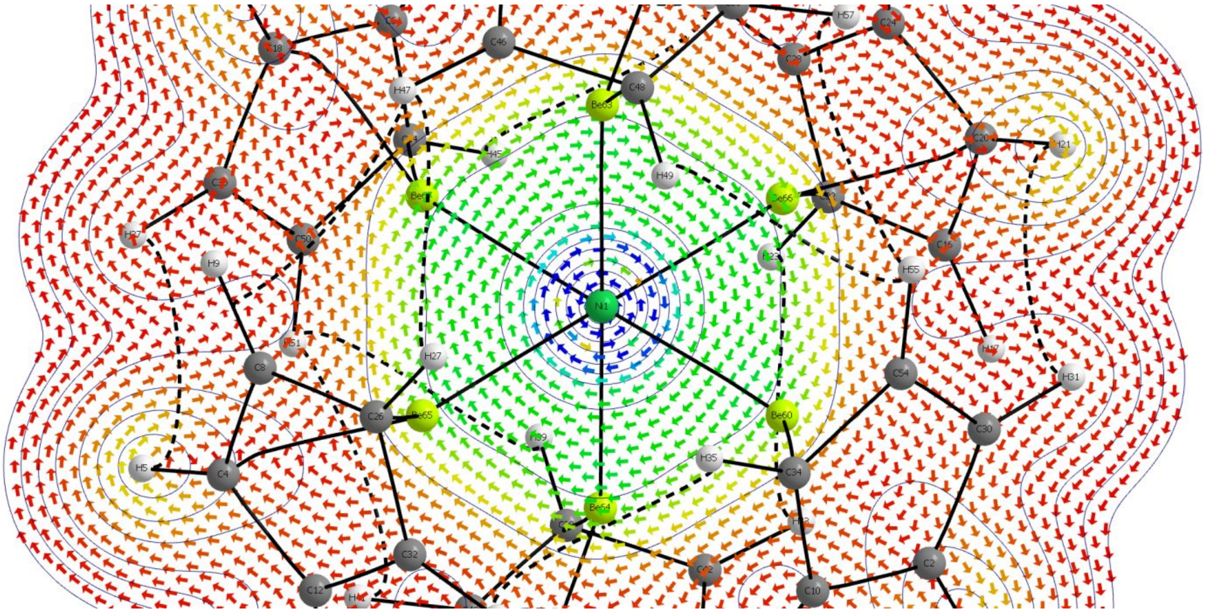

Figure S41: Ring current plot for **1**. A clockwise (diamagnetic) current can clearly be seen. Counter-clockwise currents towards the inner rim of the diamagnetic region can also be seen. This is analogous to benzene (fig. S40). The blue to red colour scale indicates strong (J(r) ≥ 0.001 a.u.) to weak currents (J(r) = 0.0000)

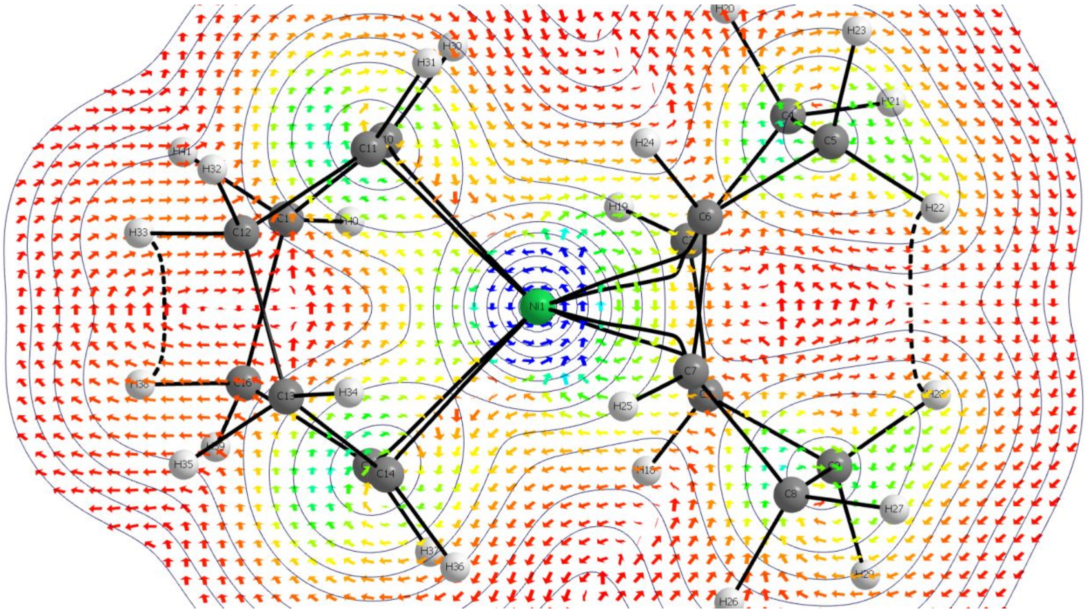

Figure S42: Ring current plot for $Ni(COD)_2$. Only local paramagnetic currents can be seen surrounding the Ni centre, which is typical of a metal. The blue to red colour scale indicates strong (J(r) ≥ 0.001 a.u.) to weak currents (J(r) = 0.0000)

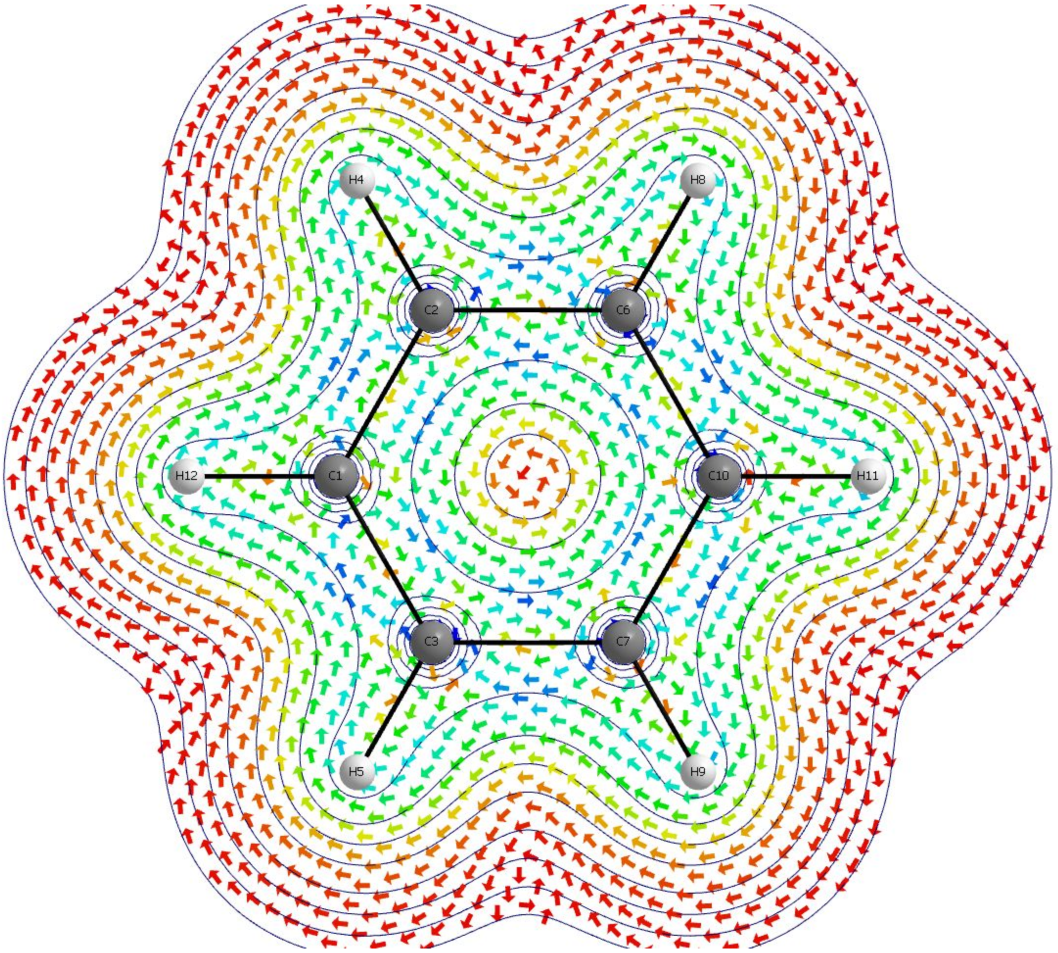

Figure S43: Ring current plot for benzene. A clockwise (diamagnetic) current can clearly be seen around the outside of the C_6 ring. Counter-clockwise currents around the inner rim of the C_6 ring can also be seen. The blue to red colour scale indicates strong ($J(r) \geq 0.001$ a.u.) to weak currents ($J(r) = 0.0000$)

Anisotropy of Induced Current Density (ACID) Analysis:

Anisotropy of Induced Current Density (ACID) was performed with the code provided to us by the research group of Prof. Herges, from the wavefunction generated by Gaussian 16 (CSGT method) [30,31].

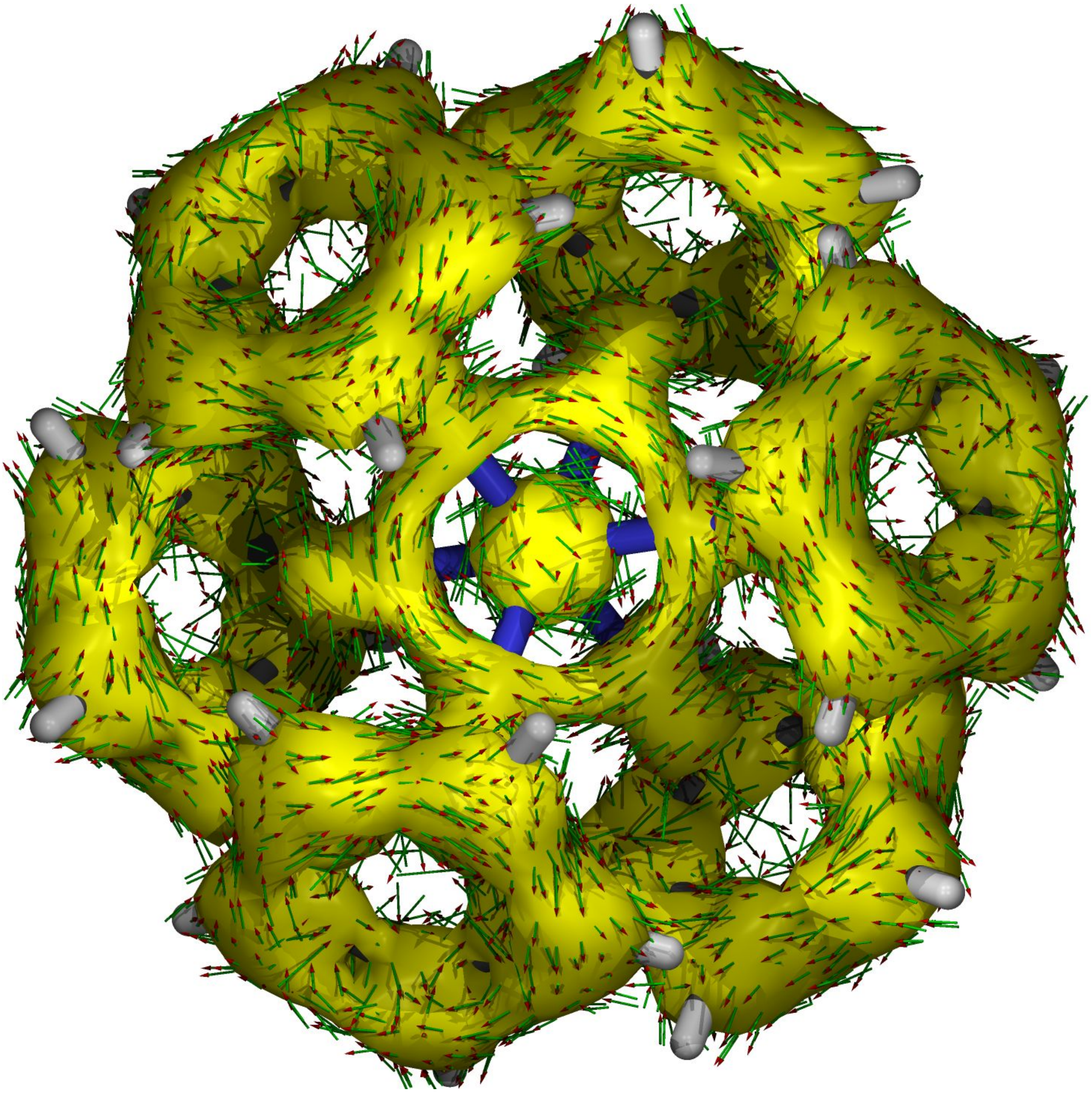

Figure S44: ACID plot for **1** at 0.05 a.u. isosurface value. The clockwise direction of the induced current (illustrated by red arrow heads) indicates aromatic delocalisation.

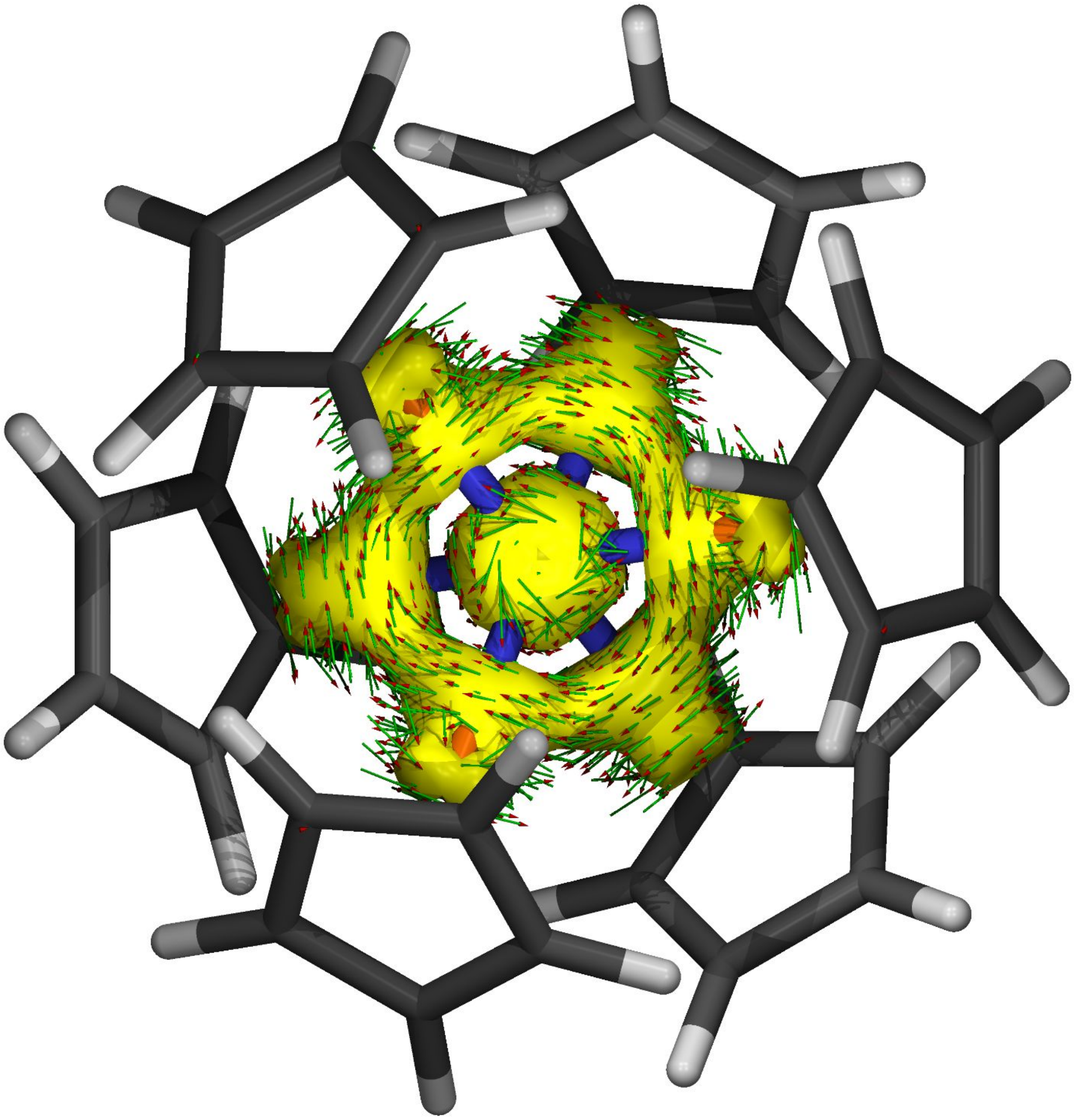

Figure S45: ACID plot of the $NiBe_6$ bonding orbitals of **1** at 0.05 a.u. isosurface value. The clockwise direction of the induced current (illustrated by red arrow heads) indicates aromatic delocalisation.

References:

(1) Boronski, J. T.; Crumpton, A. E.; Wales, L. L.; Aldridge, S. Diberyllocene, a Stable Compound of Be(I) with a Be–Be Bond. *Science.* **2023**, *380* (6650), 1147–1149. https://doi.org/10.1126/science.adh4419.

(2) Buchner, M. R.; Müller, M. Handling Beryllium, the Safe Way. *ACS Chem. Heal. Saf.* **2023**, *30* (2), 36–43. https://doi.org/10.1021/acs.chas.3c00003.

(3) Cosier, J.; Glazer, A. M. A Nitrogen-Gas-Stream Cryostat for General X-Ray Diffraction Studies. *J. Appl. Crystallogr.* **1986**, *19* (2), 105–107. https://doi.org/10.1107/S0021889886089835.

(4) Agilent Technologies. CrysAlisPro.

(5) Sheldrick, G. M. Crystal Structure Refinement with SHELXL. *Acta Crystallogr. Sect. C Struct. Chem.* **2015**, *71* (1), 3–8. https://doi.org/10.1107/S2053229614024218.

(6) Sheldrick, G. M. SHELXT – Integrated Space-Group and Crystal-Structure Determination. *Acta Crystallogr. Sect. A Found. Adv.* **2015**, *71* (1), 3–8. https://doi.org/10.1107/S2053273314026370.

(7) Dolomanov, O. V.; Bourhis, L. J.; Gildea, R. J.; Howard, J. A. K.; Puschmann, H. OLEX2 : A Complete Structure Solution, Refinement and Analysis Program. *J. Appl. Crystallogr.* **2009**, *42* (2), 339–341. https://doi.org/10.1107/S0021889808042726.

(8) Neese, F. The ORCA Program System. *WIREs Comput. Mol. Sci.* **2012**, *2* (1), 73–78. https://doi.org/10.1002/wcms.81.

(9) Neese, F. Software Update: The ORCA Program System, Version 4.0. *WIREs Comput. Mol. Sci.* **2018**, *8* (1). https://doi.org/10.1002/wcms.1327.

(10) Mardirossian, N.; Head-Gordon, M. ΩB97X-V: A 10-Parameter, Range-Separated Hybrid, Generalized Gradient Approximation Density Functional with Nonlocal Correlation, Designed by a Survival-of-the-Fittest Strategy. *Phys. Chem. Chem. Phys.* **2014**, *16* (21), 9904. https://doi.org/10.1039/c3cp54374a.

(11) Najibi, A.; Goerigk, L. DFT-D4 Counterparts of Leading Meta-Generalized-Gradient Approximation and Hybrid Density Functionals for Energetics and Geometries. *J. Comput. Chem.* **2020**, *41* (30), 2562–2572. https://doi.org/10.1002/jcc.26411.

(12) Weigend, F.; Ahlrichs, R. Balanced Basis Sets of Split Valence, Triple Zeta Valence and Quadruple Zeta Valence Quality for H to Rn: Design and Assessment of Accuracy. *Phys. Chem. Chem. Phys.* **2005**, *7* (18), 3297. https://doi.org/10.1039/b508541a.

(13) Weigend, F. Accurate Coulomb-Fitting Basis Sets for H to Rn. *Phys. Chem. Chem. Phys.* **2006**, *8* (9), 1057. https://doi.org/10.1039/b515623h.

(14) Caldeweyher, E.; Mewes, J.-M.; Ehlert, S.; Grimme, S. Extension and Evaluation of the D4 London-

Dispersion Model for Periodic Systems. *Phys. Chem. Chem. Phys.* **2020**, *22* (16), 8499–8512. https://doi.org/10.1039/D0CP00502A.

(15) Grimme, S.; Hansen, A.; Ehlert, S.; Mewes, J.-M. R2SCAN-3c: A "Swiss Army Knife" Composite Electronic-Structure Method. *J. Chem. Phys.* **2021**, *154* (6). https://doi.org/10.1063/5.0040021.

(16) Furness, J. W.; Kaplan, A. D.; Ning, J.; Perdew, J. P.; Sun, J. Accurate and Numerically Efficient r 2 SCAN Meta-Generalized Gradient Approximation. *J. Phys. Chem. Lett.* **2020**, *11* (19), 8208–8215. https://doi.org/10.1021/acs.jpclett.0c02405.

(17) Kruse, H.; Grimme, S. A Geometrical Correction for the Inter- and Intra-Molecular Basis Set Superposition Error in Hartree-Fock and Density Functional Theory Calculations for Large Systems. *J. Chem. Phys.* **2012**, *136* (15). https://doi.org/10.1063/1.3700154.

(18) Frisch, M. J.; Trucks, G. W.; Schlegel, H. B.; Scuseria, G. E.; Robb, M. A.; Cheeseman, J. R.; Scalmani, G.; Barone, V.; Petersson, G. A.; Nakatsuji, H.; Li, X.; Caricato, M.; Marenich, A. V.; Bloino, J.; Janesko, B. G.; Gomperts, R.; Mennucci, B.; Hratch, D. J. Gaussian 16. Gaussian Inc.: Walingford CT 2016.

(19) Chai, J.-D.; Head-Gordon, M. Long-Range Corrected Hybrid Density Functionals with Damped Atom–Atom Dispersion Corrections. *Phys. Chem. Chem. Phys.* **2008**, *10* (44), 6615. https://doi.org/10.1039/b810189b.

(20) Boronski, J. T.; Crumpton, A. E.; Aldridge. A Hexavalent Nickel Complex: Xyz Coordinates of Optimised Structures. *Dryad* **2023**.

(21) Todd, K. A. AIMAll (Version 19.10.12). TK Gristmill Software: Overland Park KS 2019. aim.tkgristmill.com.

(22) Lu, T.; Chen, F. Multiwfn: A Multifunctional Wavefunction Analyzer. *J. Comput. Chem.* **2012**, *33* (5), 580–592. https://doi.org/10.1002/jcc.22885.

(23) Silvi, B.; Savin, A. Classification of Chemical Bonds Based on Topological Analysis of Electron Localization Functions. *Nature* **1994**, *371* (6499), 683–686. https://doi.org/10.1038/371683a0.

(24) Jacobsen, H. Localized-Orbital Locator (LOL) Profiles of Chemical Bonding. *Can. J. Chem.* **2008**, *86* (7), 695–702. https://doi.org/10.1139/v08-052.

(25) Michalski, M.; Gordon, A. J.; Berski, S. Topological Analysis of the Electron Localisation Function (ELF) Applied to the Electronic Structure of Oxaziridine: The Nature of N-O Bond. *Struct. Chem.* **2019**, *30* (6), 2181–2189. https://doi.org/10.1007/s11224-019-01407-9.

(26) Glendening, E. D.; Badenhoop, J. K.; Reed, A. E.; Carpenter, J. E.; A., B. J.; Morales, C. M.; Karafiloglou, P.; Landis, C. R.; Weinhold, F. NBO 7.0. Madison, WI 2018.

(27) Zubarev, D. Y.; Boldyrev, A. I. "Developing Paradigms of Chemical Bonding: Adaptive Natural

Density Partitioning. *Phys. Chem. Chem. Phys.* **2008**, *10* (34), 5207. https://doi.org/10.1039/b804083d.

(28) Tkachenko, N. V.; Boldyrev, A. I. Chemical Bonding Analysis of Excited States Using the Adaptive Natural Density Partitioning Method. *Phys. Chem. Chem. Phys.* **2019**, *21* (18), 9590–9596. https://doi.org/10.1039/C9CP00379G.

(29) Szczepanik, D. W.; Andrzejak, M.; Dominikowska, J.; Pawełek, B.; Krygowski, T. M.; Szatylowicz, H.; Solà, M. The Electron Density of Delocalized Bonds (EDDB) Applied for Quantifying Aromaticity. *Phys. Chem. Chem. Phys.* **2017**, *19* (42), 28970–28981. https://doi.org/10.1039/C7CP06114E.

(30) Ab Initio Calculation of the Anisotropy Effect of Multiple Bonds and the Ring Current Effect of Arenes—Application in Conformational and Configurational Analysis. *J. Chem. Soc. Perkin Trans. 2* **2001**, No. 10, 1893–1898. https://doi.org/10.1039/b009809o.

(31) Geuenich, D.; Hess, K.; Köhler, F.; Herges, R. Anisotropy of the Induced Current Density (ACID), a General Method To Quantify and Visualize Electronic Delocalization. *Chem. Rev.* **2005**, *105* (10), 3758–3772. https://doi.org/10.1021/cr0300901.

(32) Herges, R.; Geuenich, D. Delocalization of Electrons in Molecules. *J. Phys. Chem. A* **2001**, *105* (13), 3214–3220. https://doi.org/10.1021/jp0034426.
